# GABA_A_ Receptors Containing ρ1 Subunits Contribute to *In Vivo* Effects of Ethanol in Mice

**DOI:** 10.1371/journal.pone.0085525

**Published:** 2014-01-16

**Authors:** Yuri A. Blednov, Jillian M. Benavidez, Mendy Black, Courtney R. Leiter, Elizabeth Osterndorff-Kahanek, David Johnson, Cecilia M. Borghese, Jane R. Hanrahan, Graham A. R. Johnston, Mary Chebib, R. Adron Harris

**Affiliations:** 1 Waggoner Center for Alcohol and Addiction Research, The University of Texas at Austin, Austin, Texas, United States of America; 2 Faculty of Pharmacy, The University of Sydney, Sydney NSW, Australia; 3 Department of Pharmacology, The University of Sydney, Sydney NSW, Australia; Nathan Kline Institute for Psychiatric Research and New York School of Medicine, United States of America

## Abstract

GABA_A_ receptors consisting of ρ1, ρ2, or ρ3 subunits in homo- or hetero-pentamers have been studied mainly in retina but are detected in many brain regions. Receptors formed from ρ1 are inhibited by low ethanol concentrations, and family-based association analyses have linked ρ subunit genes with alcohol dependence. We determined if genetic deletion of ρ1 in mice altered *in vivo* ethanol effects. Null mutant male mice showed reduced ethanol consumption and preference in a two-bottle choice test with no differences in preference for saccharin or quinine. Null mutant mice of both sexes demonstrated longer duration of ethanol-induced loss of righting reflex (LORR), and males were more sensitive to ethanol-induced motor sedation. In contrast, ρ1 null mice showed faster recovery from acute motor incoordination produced by ethanol. Null mutant females were less sensitive to ethanol-induced development of conditioned taste aversion. Measurement of mRNA levels in cerebellum showed that deletion of ρ1 did not change expression of ρ2, α2, or α6 GABA_A_ receptor subunits. (*S*)-4-amino-cyclopent-1-enyl butylphosphinic acid (“ρ1” antagonist), when administered to wild type mice, mimicked the changes that ethanol induced in ρ1 null mice (LORR and rotarod tests), but the ρ1 antagonist did not produce these effects in ρ1 null mice. In contrast, (*R*)-4-amino-cyclopent-1-enyl butylphosphinic acid (“ρ2” antagonist) did not change ethanol actions in wild type but produced effects in mice lacking ρ1 that were opposite of the effects of deleting (or inhibiting) ρ1. These results suggest that ρ1 has a predominant role in two *in vivo* effects of ethanol, and a role for ρ2 may be revealed when ρ1 is deleted. We also found that ethanol produces similar inhibition of function of recombinant ρ1 and ρ2 receptors. These data indicate that ethanol action on GABA_A_ receptors containing ρ1/ρ2 subunits may be important for specific effects of ethanol *in vivo*.

## Introduction

Ionotropic γ-aminobutyric acid A (GABA_A_) receptors represent the major inhibitory class of neurotransmitter receptors in the mammalian brain. They are pentameric in structure, with five subunits forming an ion pore. Eight classes of GABA_A_ receptor subunits have been described to date (α1–6, β1–3, γ1–3, δ, ε, θ, π, ρ1–3), allowing for extensive heterogeneity in receptor subunit composition across neuronal cell types and brain regions. However, most native GABA_A_ receptors are thought to consist of two α, two β, and one γ or δ subunit.

GABA_A_ receptors mediate a number of pharmacological effects, including sedation/hypnosis, anxiolysis, and anesthesia, by drugs such as barbiturates, benzodiazepines, neuroactive steroids, and intravenous anesthetics. There is also considerable evidence that ethanol enhances the function of GABA_A_ receptors, but we are only beginning to elucidate the specific roles of each receptor subtype and its component subunits in ethanol-induced behavior modification [1]–[5].

Some GABA_A_ receptors can be formed from homo- or hetero-pentamers composed of ρ1, ρ2, or ρ3 subunits (previously termed GABA_C_ receptors). They have been studied in the retina where they are expressed in bipolar and horizontal cells, but they are also present in many brain regions [6]. Elimination of ρ1 subunit expression leads to a complete loss of GABA_A_ ρ receptor function in the retina [7]. As a consequence, retinal bipolar cells in GABA_A_ ρ1 null mice lack GABA_A_ receptor-mediated feedback currents without compensatory changes in other inhibitory inputs [8], and related components of the electroretinogram are strongly enhanced in these mice [7]. In addition, there is evidence for functional GABA receptors containing ρ subunits in the spinal cord, superior colliculus, pituitary, and the gut and their involvement in vision, aspects of memory, and sleep-waking [9]. There are three distinctive functional characteristics that are unique to the homomeric GABA_A_ ρ receptor: long mean opening time of the channel, low conductance, and low rate of desensitization. The mean open time of the channel ranges from 150 to 200 ms, which is more than five-fold longer than that of other GABA_A_ subunits [10].

There is some evidence for co-assembly of ρ1 subunits in the spinal cord and brain stem with other GABA_A_ receptor subunits, suggesting that they form functional heteromeric complexes [11]–[13]. Glycine, taurine, and β-alanine [14]–[16] have been shown to activate GABA_A_ ρ receptors at concentrations that may be reached in the synapse, indicating that these amino acids might modulate synaptic transmission across GABAergic synapses.

The response of GABA_A_ ρ receptors to ethanol is distinct from classical GABA_A_ receptors in that homomeric receptors formed from ρ1 are inhibited by low concentrations of ethanol [17]. Unexpectedly, family-based association analyses have linked the ρ subunit genes with alcohol dependence [18]. To our knowledge, no *in vivo* studies have examined this linkage; consequently, we determined if genetic deletion of the ρ1 subunit in mice [7] would alter ethanol responses.

## Materials and Methods

### Ethics statement

All experiments were approved by the Institutional Animal Care and Use Committee at The University of Texas (#AUP 2013-00061) and were conducted in accordance with National Institutes of Health guidelines with regard to the care and use of animals in research.

### Animals

Mice lacking the ρ1 subunit of the GABA_A_ receptor - B6;129S4-*Gabrr1^tm1Llu^*/J (Stock # 010535) were purchased from Jackson Laboratories (Bar Harbor, ME), and the colony was maintained as heterozygous breeding without changing the genetic background. After weaning, mice were housed in the Animal Resources Center at The University of Texas with *ad libitum* access to rodent chow and water with 12-h light/dark cycles (lights on at 7:00 AM). All mice were between 8 and 12 weeks of age. Both male and female mice were used. Each mouse was used for only one experiment, and all mice were ethanol-naive at the start of each experiment.

### Ethanol preference drinking, 24-hour access

A two-bottle choice protocol was carried out as previously described [19]. Briefly, mice were allowed to acclimate for 1 week to individual housing. Two drinking tubes were continuously available to each mouse, and tubes were weighed daily. One tube always contained water. Food was available *ad libitum*, and mice were weighed every 4 days. After 4 days of water consumption (both tubes), mice were offered 3% ethanol (v/v) versus water for 4 days. Tube positions were changed daily to control for position preferences. Quantity of ethanol consumed (g/kg body weight/24 hours) was calculated for each mouse, and these values were averaged for every concentration of ethanol. Immediately following 3% ethanol, a choice between 6% (v/v) ethanol and water was offered for 4 days, then 9% (v/v) ethanol for 4 days, then 12% (v/v) ethanol for 4 days, then 15% (v/v) ethanol for 4 days and finally, 18% (v/v) ethanol for 4 days. Throughout the experiment, evaporation/spillage estimates were calculated daily from two bottles placed in an empty cage, one containing water and the other containing the appropriate ethanol solution.

### Preference for non-ethanol tastants, 24-hour access

Mice were also tested for saccharin and quinine consumption. One tube always contained water, and the other contained the tastant solution. Mice were serially offered saccharin (0.0165%, 0.033% and 0.066%) and quinine hemisulfate (0.03 and 0.06 mM), and intake was calculated. Each concentration was offered for 4 days, with bottle position changed daily. For each tastant, the low concentration was always presented first, followed by the higher concentration. Between tastant testing, mice had access to two bottles with water for two weeks.

### Ethanol drinking - limited access drinking in the dark phase (one-bottle DID)

Another approach for consumption of ethanol (15% solution) was recently described under conditions of limited access, which achieves pharmacologically significant levels of ethanol drinking [20]. Briefly, starting at 3 hours after lights off, the water bottles were replaced with a bottle containing a 15% ethanol solution. The ethanol bottle remained in place for either 2 (first 3 days) or 4 hours (day 4) and then was replaced with the water bottles. Other than these short periods of ethanol drinking, mice had unlimited access to water. The ethanol bottles were weighed before placement and after removal of the bottles from each experimental cage.

### Ethanol drinking - 24-hour access every other day (intermittent drinking)

During the 1970s, several studies showed that intermittent access to ethanol induced high voluntary ethanol consumption [21]–[23]. Recently Simms et al. (2008) resurrected this experimental approach and showed that it produces reproducibly high levels of voluntarily ethanol consumption in Long–Evans or Wistar rats [24]. Therefore, we assessed ethanol consumption using a paradigm adapted from Wise (1973) [23] and Simms et al. (2008) [24], employing intermittent access to 15% ethanol. Animals were given access to one bottle of ethanol and one bottle of water during 24-hour sessions every other day. The placement of the ethanol bottle was alternated with each ethanol drinking session to control for side preferences.

### Conditioned taste aversion (CTA)

Subjects were adapted to a water-restriction schedule (2 hours of water per day) over a 7-day period. At 48-hour intervals over the next 10 days (days 1, 3, 5, 7, 9 and 11), all mice received 1-hour access to a solution of saccharin (0.15% w/v sodium saccharin in tap water). Immediately after 1-hour access to saccharin, mice received injections of saline or ethanol (2.5 g/kg) (days 1, 3, 5, 7 and 9). All mice also received 30-minute access to tap water 5 hours after each saccharin-access period to prevent dehydration (days 1, 3, 5, 7 and 9). On intervening days, mice had 2-hour continuous access to water at standard times in the morning (days 2, 4, 6, 8 and 10). Reduced consumption of the saccharin solution is used as a measure of CTA.

To measure aversion extinction, all mice were given access to both water and saccharin *ad libitum* on the next day after the last measure in the CTA procedure. Intake of each fluid was measured daily during 6 days, and saccharin preference ratios were calculated by dividing the amount of saccharin solution consumed by the total amount of fluid consumed. After 6 days of two-bottle choice, mice had access to only one bottle of water for two weeks, and then the two-bottle choice experiment with free access to water and saccharin was repeated again. In total, three rounds of two-bottle choice drinking for 6 days each with two-week breaks were carried out.

### Conditioned place preference

The conditioned place preference protocol was carried out as previously described [19]. Four identical acrylic boxes (30×15×15 cm) were separately enclosed in ventilated, light, and sound-attenuating chambers (Med Associates, St. Albans, VT). Each box has two compartments separated by a wall with a door. The two compartments each have a different type of floor (either bars set in a grid or small round holes). Infrared light sources and photodetectors were mounted opposite each other at 2.5-cm intervals along the length of each box, 2.2 cm above the floor. Occlusion of the infrared light beams was used to measure general activity and location of the animal (left or right) within the box. Total activity counts and location of the animal (left or right compartment) within the box were recorded by computer. The floors and the inside of the boxes were wiped with water, and the litter paper beneath the floors was changed between animals. The main principles of the conditioned place preference procedure have been described earlier [25]. Ethanol was used at a dose of 2.0 g/kg (i.p.). During the 10 days of extinction, all mice received 5 daily, non-reinforced exposures to each of the conditioned and unconditioned stimulus cues separately (5 minutes each). After the last day of extinction, mice were exposed to a 30-minute preference test with full access to both floor types.

### Ethanol-induced acute withdrawal

Mice were scored for handling-induced convulsion (HIC) severity 30 minutes before and immediately before i.p. ethanol administration. The two pre-drug baseline scores were averaged. A dose of 4.0 g/kg of ethanol in saline was injected i.p., and the HIC score was tested every hour until the HIC level reached base-line. Acute withdrawal was quantified as the area under the curve but above the pre-drug level [26]. Briefly, each mouse is picked up gently by the tail and, if necessary, gently rotated 180°, and the HIC is scored as follows: 5, tonic-clonic convulsion when lifted; 4, tonic convulsion when lifted; 3, tonic-clonic convulsion after a gentle spin; 2, no convulsion when lifted, but tonic convulsion elicited by a gentle spin; 1, facial grimace only after a gentle spin; 0, no convulsion.

### Startle reflex

Acoustic startle responses were measured using SR-LAB test stations and software (San Diego Instruments, San Diego, CA). Startle responses were recorded as described previously [27]. Briefly, test sessions began by placing the mouse in a Plexiglas holding cylinder for a 5-minute acclimation period. Over the next 8 minutes, mice were presented with each of seven trial types across five discrete blocks of trials for a total of 35 trials. The inter-trial interval was 10–20 s. One trial measured the response to no stimulus (baseline movement). The other six trials measured the response to a startle stimulus alone, consisting of a 40 ms sound burst of 90, 95, 100, 105, 110 or 115 dB. Startle response amplitude was measured every 1 ms over a 65-ms period beginning at the onset of the startle stimulus. The six trial types were presented in pseudorandom order such that each trial type was presented once within a block of six trials. The maximum startle amplitude (Vmax) over this sampling period was taken as the dependent variable. A background noise level of 70 dB was maintained over the duration of the test session.

### Loss of righting reflex (LORR)

Sensitivity to depressant effects of ethanol (3.8 g/kg) and other drugs such as flurazepam (225 mg/kg), pentobarbital (50 mg/kg), and ketamine (175 mg/kg) were determined using the standard duration of LORR (sleep time) assay in mice. When mice became ataxic, they were placed in the supine position in V-shaped plastic troughs until they were able to right themselves three times within 30 s. Sleep time was defined as the time from being placed in the supine position until they regained their righting reflex. When measuring effects of ρ1/ρ2 antagonists on duration of LORR, the ethanol and ketamine doses used were 3.4 g/kg and 150 mg/kg, respectively.

### Rotarod

Mice were trained on a fixed speed rotarod (Economex; Columbus Instruments, Columbus, OH) at 5 rpm, and training was considered complete when mice were able to remain on the rotarod for 60 s. Every 15 minutes after injection of ethanol (2.0 g/kg i.p.), each mouse was placed back on the rotarod and latency to fall was measured until the mouse was able to stay on the rotarod for 60 s.

### Elevated plus maze

Mice were evaluated for basal anxiety-related behaviors as well as ethanol-induced anxiolysis using the elevated plus maze as described previously [28]. Mice were transported to the testing room 1 day prior to testing. Animals were tested between 10:00 and 12:00 AM under ambient room light. Mice were weighed and injected with ethanol (1.0 g/kg and 1.25 g/kg, i.p.) or saline 10 minutes prior to testing. Each mouse was placed on the central platform of the maze facing an open arm. Mice were allowed to freely explore the maze for 5 minutes during which the following measurements were manually recorded: number of open arm entries, number of closed arm entries, total number of entries, time spent in open arms, and time spent in closed arms. A mouse was considered to be on the central platform or any arm when all four paws were within its perimeter.

### Motor activity testing

Locomotor activity was measured in standard mouse cages using the Opto-microvarimex animal activity meter (Columbus Instruments, Columbus, OH). Activity was monitored by 6 light beams placed along the width of the cage at 2.5 cm intervals, 1.5 cm above the floor. Each cage had bedding and food and was covered by a heavy plastic lid with holes for ventilation. At the end of the first day, mice were removed from the home cages, weighed, and injected with saline (i.p.). After saline administration, mice were placed immediately in individual experimental cages, and activity was monitored every 5 minutes for 15 minutes. This procedure was repeated for 3 consecutive days. During this period of time, each mouse was pre-habituated to handling, stress of transference to experimental cage, and to saline injection. During the entire experimental period (5 days), each mouse had the same experimental environment (familiar cage with the same bedding and food). On day 4, mice received ethanol injections at a dose of 1.0 g/kg and, on day 5, mice received 1.5 g/kg ethanol; control mice received saline injections. In the control group, motor responses to saline on days 4 and 5 were similar to their motor responses on day 3. Therefore, motor activity of ethanol-treated mice on days 4 and 5 was compared with their motor response after saline injection on day 3.

### Ethanol metabolism

Animals were given a single dose of ethanol (4.0 g/kg, i.p.), and blood samples were taken from the retro-orbital sinus 30, 60, 120, 180, and 240 minutes after injection. Blood ethanol concentration (BEC) values, expressed as mg ethanol per ml blood, were determined spectrophotometrically by an enzyme assay [29].

### Missteps (foot-slips) test

Sensorimotor asymmetry was assessed using Columbus Instruments' new foot misplacement apparatus that consists of a set of two stainless steel horizontal ladders (94 cm long, 20 cm wide, 48 cm high, with 4 cm space between two ladder beams) (Columbus Instruments, Columbus, OH). These horizontal ladders were divided into a safety end with a dark compartment and a shock end that produces an electric shock from the shock generator. For training, each animal was placed initially on the shock end. If the animal missed the ladder and touched the metal plate, which is located below the horizontal ladder, it received a foot shock and moved toward the safety end. After repeated missteps, the mice eventually stayed at the safety end. This training session lasted no longer than 5 minutes. Twenty-four hours later, an actual test was started, and each animal received a control injection of saline and was again placed on the shock end while the shock generator was turned on. The number of missteps was counted automatically by detecting the change of resistance between the ladder and the metal plate each time the animal missed one of the rungs of the ladder and touched the metal plate below as it moved toward the safety end. Two hours later, each animal received an injection of ethanol and was placed on the ladder again. Different doses of ethanol (1.0 and 1.5 g/kg) were tested on different days. For each animal, the test sessions with saline or ethanol were repeated twice during a 2–3 minute period 5 minutes after injection.

### Grip strength test

Grip strength was assessed using a grip strength meter consisting of horizontal forelimb mesh (Columbus Instruments, Columbus, OH). Three successful forelimb strength measurements within 2 minutes were recorded and normalized to body weight as previously described [30].

### RT-*q*PCR measurement of GABA_A_ receptor subunits in cerebellum

Cerebellar tissue from 20 wild type (n = 11 females, n = 9 males) and 18 ρ1 null (n = 10 females, n = 8 males) mice were dissected, flash-frozen in liquid N_2_, and stored at −80°C. Total RNA was isolated using the MagMax-96 for microarrays kit (Ambion, Austin, TX). RNA concentration and purity were determined by UV spectrometry (Nanodrop; Thermo Scientific, Wilmington, DE), and overall RNA integrity was assessed using a 2200 TapeStation (Agilent Technologies, Santa Clara, CA). Each RNA sample was reverse transcribed into cDNA using a High-Capacity cDNA Reverse Transcription Kit (Applied Biosystems, Foster City, CA). qPCR was performed in triplicate for 90 ng of each cDNA using SsoAdvanced Universal Probes Supermix, according to manufacturer's instructions (Bio-rad, Hercules, CA). FAM-labeled TaqMan Gene Expression Assays (Applied Biosystems) were used to amplify *Gabrr1* (Mm01212386_m1), *Gabrr2* (Mm00433510_m1), *Gabra2* (Mm00433435_m1), *Gabra6* (Mm01227754_m1), and *Gusb* (Mm01197698_m1). RT-qPCR results were imported into qBase+ software, version 2.5 (Biogazelle, Gent, BE), where the single threshold Cq determination and ΔΔCt methods were used [31]. Data were normalized to the reference gene *Gusb*, which demonstrated minimal variation among mean sample Cq values (range of 1.6). Wild type and null mutant groups were compared using an unpaired *t*-test.

### Electrophysiology in xenopus oocytes

The rho subunits used for expression in oocytes were from human origin. Alignment with the mouse subunits showed high identity between the mature human and mouse proteins (94% for rho1 and 91% for rho2); the homology was even greater for the transmembrane domains (100% for rho1 and 96% for rho2), which are critical for ethanol effects. However, one of the amino acids that differ between mouse and human rho2 is critical for picrotoxin/picrotoxinine inhibition (threonine in 6′ position in human rho2, methionine in mouse rho2) [32], [33]. To determine if this amino acid could influence the effect of ethanol (which is also inhibitory), we studied human rho1(T6′M) expressed in oocytes (we introduced this mutation in rho1 instead of rho2 because rho2 is considerably more difficult to express). When we applied 200 mM ethanol in the presence of an EC_20_ GABA concentration, the inhibitory effect was the same in wild type and T6′M mutant. The high expression of human subunits and high degree of homology with mouse subunits, especially within the critical transmembrane region, together with verifying that one of the potentially important amino acid differences between human and mouse is not involved in ethanol action, all provide strong rationale for using human subunits for expression studies.

The materials used and the procedures followed were essentially those described in Borghese et al. (2006) [34]. We will briefly describe the procedures and any differences from the original description. The cDNAs encoding the human GABA_A_ ρ1 and ρ2 subunits were in pcDNA1 and pcDNA3.1 plasmids, respectively, and were kindly provided by Dr. Garry C. Cutting. The coding sequence for ρ2 was excised using Eco RI and XhoI, and inserted into the pGEMHE vector after cutting it with Eco RI and HindIII. The ρ2-pGEMHE construct was linearized with PstI and used as a template for the synthesis *in vitro* of capped RNA (mMessage mMachine, Ambion, Life Technologies, Grand Island, NY).

*Xenopus laevis* oocytes were manually isolated from a surgically removed portion of ovary. Oocytes were treated with collagenase for 10 minutes, and then placed in sterile Modified Barth's Solution (MBS, composition: 88 mM NaCl, 1 mM KCl, 2.4 mM NaHCO_3_, 10 mM HEPES, 0.82 mM MgSO_4_, 0.33 mM Ca(NO_3_)_2_, 0.91 mM CaCl_2_, adjusted to pH 7.5), supplemented with 10,000 units penicillin, 50 mg gentamicin, 90 mg theophylline and 220 mg sodium pyruvate per liter (Incubation medium). Oocytes were either injected into the nucleus with 50 nl of a solution containing cDNA encoding GABA_A_ ρ1 (1.5 ng/oocyte), or into the equator with 40 nl of a solution containing cRNA encoding GABA_A_ ρ2 (20–40 ng/oocyte). The injected oocytes were kept at 19°C in Incubation medium.

Recordings were carried out 4–8 days after injection. The oocytes were placed in a rectangular chamber (approximately 100 µl) and continuously perfused with MBS buffer (2 ml/minute) at room temperature (24°C). The whole-cell voltage clamp at −70 mV was achieved through two glass electrodes (1.5–10 MΩ) filled with 3 M KCl, using a Warner Instruments (Hamden, CT) oocyte clamp, model OC-725C.

All drugs were applied by bath-perfusion, and all solutions were prepared on the day of the experiment. The concentration response curves (CRCs) were obtained with increasing concentrations of GABA, applied for 30–60 s at intervals ranging from 10 to 15 minutes. From these CRCs, the concentration evoking a half-maximal response (EC_50_) was calculated, along with the Hill coefficient (see the Statistical Analysis section). To study the ethanol (30–200 mM) modulation of GABA currents, the GABA concentration equivalent to EC_50_ was determined after 1–10 mM GABA gave the maximal current. A washout of 10 minutes was observed in-between all GABA applications, except after maximal GABA concentration (15 minutes). After two applications of EC_50_ GABA, ethanol was pre-applied for 1 minute and then co-applied with GABA for 60–90 s. EC_50_ GABA was applied again, and the procedure repeated with another ethanol concentration. All experiments shown include data obtained from oocytes taken from at least two different frogs. All oocytes that presented a maximal current >20 µA were discarded.

Nonlinear regression analysis was performed with Prism (GraphPad Software Inc., San Diego, CA). Agonist responses in each cell were normalized to the maximal current that could be elicited by GABA. Percent change was calculated as the percentage change from the control response to EC_50_ GABA in the presence of ethanol. Pooled data are represented as mean ± standard error.

### Rationale for the *in vivo* tests

Two-bottle choice (continuous, 24-hour access) is the most widely used test of ethanol preference, and intake allows measurement of voluntary consumption. It appears to be related to other measures of ethanol reward [35]. Other tests for ethanol intake produce high levels of ethanol consumption by limiting access to ethanol or allowing only intermittent access to ethanol. Because ethanol produces taste responses (sweet and bitter), it is critical to analyze the sensitivity of the genotypes to bitter (quinine solutions) and sweet (saccharin solutions) tastes to determine if changes in ethanol consumption are secondary to changes in taste [36]. Conditioned taste aversion is used as the index of aversive properties to ethanol, and the response in this test is negatively correlated with voluntary ethanol intake [35], whereas conditioned place preference is broadly used for evaluation of rewarding properties of drugs of abuse. Duration of LORR measures the anesthetic or sedative activities of ethanol, and for some mutant mice it is negatively correlated with voluntary ethanol consumption [37]. Acute ethanol withdrawal shows sensitivity to the development of ethanol physical dependence and also negatively correlates with ethanol intake in the two-bottle choice paradigm [38]. The rotarod test measures an aspect of motor incoordination as well as recovery from acute ethanol intoxication. Because ataxia is a complex phenomenon [39], we measured some simple responses related to ataxia such as missteps and grip strength. The behaviors in the elevated-plus maze, as well as in open field tests, serve as an indicator of anxiety-related phenotypes and response to acute stress, behaviors that are regulated by GABAergic systems. For most of these tests, ethanol effects are changed after deletion of different subunits of GABA_A_ receptors [1], [5], [19]. In addition, some responses related to glycine receptor function were also evaluated. Because changes in glycine receptor function are accompanied by changes in acoustic startle response [27], we studied this behavior in ρ1 null mice. Recently we showed that different genetically-engineered mice with impairment of glycine function consistently demonstrated increased duration of LORR induced by ketamine [40]. Therefore, ketamine-induced LORR was also explored in ρ1 null mice.

### Drug injection

All injectable ethanol (Aaper Alcohol and Chemical, Shelbyville, KY) solutions were prepared in 0.9% saline (20%, v/v) and injected i.p. Flurazepam (Sigma-Aldrich, St. Louis, MO; 225.0 mg/kg, i.p.), ketamine (Sigma-Aldrich; 150 mg/kg, i.p.), and pentobarbital (Sigma/RBI, Natick, MA; 50.0 mg/kg, i.p.) were dissolved in 0.9% saline and injected at 0.01 ml/g of body weight. The ρ1/ρ2 antagonists [(*S*)-4-amino-cyclopent-1-enyl butylphosphinic acid, (S)-ACPBPA), and (*R*)-4-amino-cyclopent-1-enyl butylphosphinic acid, (R)-ACPBPA)] [41], were freshly prepared as a suspension in saline with 4–5 drops of Tween-80 and injected i.p. in wild type or ρ1 null mice in a volume of 0.1 ml/10 g of body weight 30 minutes before administration of ethanol in LORR and rotarod experiments.

### Statistical analysis

Data are reported as the mean ± S.E.M. The statistics software program GraphPad Prizm (Jandel Scientific, Costa Madre, CA) was used. Analysis of variance (two-way ANOVA or one-way ANOVA with repeated measurements with Bonferroni or Dunnett's post hoc tests, respectively) and Student's *t*-tests were carried out to evaluate differences between groups, as indicated in the figure legends.

## Results

### Ethanol consumption

**Figure 1:**
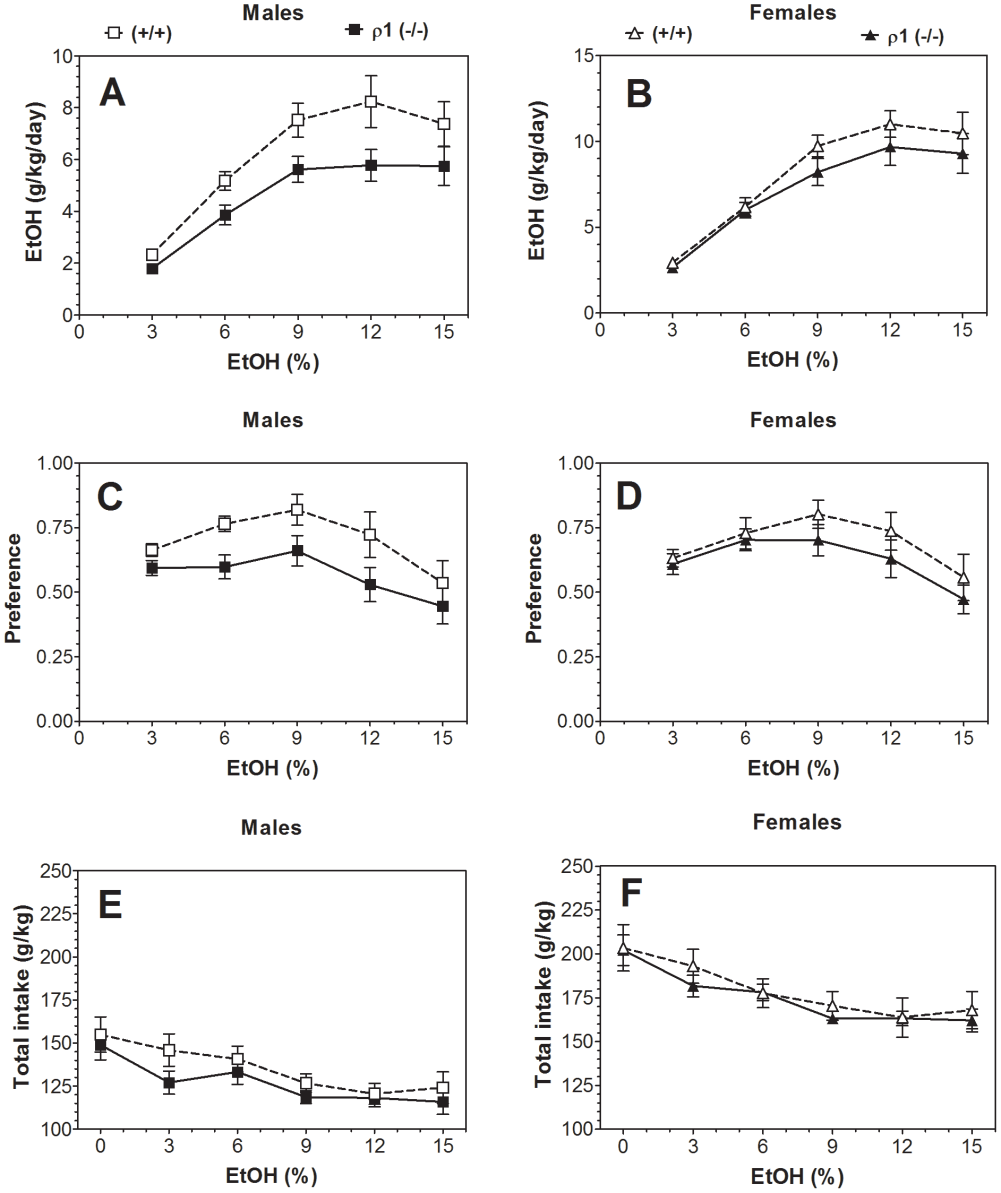
Voluntary ethanol consumption was reduced in ρ1 (−/−) male mice in 24-hour two-bottle choice paradigm. A. Ethanol consumption (g/kg/24 hours) in males. (F_1,18_ = 7.1, p<0.05, main effect of genotype; F_4,728_ = 36.2, main effect of concentration, p<0.001; no genotype x concentration interaction). B. Ethanol consumption (g/kg/24 hours) in females. (F_4,68_ = 56.2, p<0.001, main effect of concentration; no main effect of genotype or genotype x concentration interaction). C. Preference for ethanol in males. (F_1,18_ = 7.1, p<0.05, main effect of genotype; F_4,72_ = 7.8, p<0.001, main effect of concentration; no genotype x concentration interaction). D. Preference for ethanol in females. (F_4,68_ = 10.6, p<0.001, main effect of concentration; no main effect of genotype or genotype x concentration interaction). E. Total fluid intake (g/kg/24 hours) in males. (F_5,90_ = 29.9, p<0.001, main effect of concentration; no main effect of genotype or genotype x concentration interaction). F. Total fluid intake (g/kg/24 hours) in females. (F_5,75_ = 23.8, p<0.001, main effect of concentration; no main effect of genotype or genotype x concentration interaction; n = 9–10 for both genotypes and sexes). Data were analyzed by two-way ANOVA. ρ1 (−/−) = ρ1 null mice; (+/+) = wild type mice; EtOH = ethanol.

In the two-bottle choice paradigm in which mice could drink either water or an increasing series of ethanol concentrations, the amount of ethanol consumed by ρ1 null male mice was reduced compared with wild type (Fig. 1A). Null mutant male mice also demonstrated reduced preference for ethanol (Fig. 1C) as well as a decreased, but not significant, total fluid intake (Fig. 1E). In contrast, ethanol intake in female mice was similar for both genotypes (Fig. 1B). No statistically significant differences were found between ρ1 null and wild type female mice in preference for ethanol or in total amount of fluid consumed (Fig. 1D,F).

Given that ethanol intake in the continuous two-bottle choice paradigm depends strongly on taste [36], the preferences for non-ethanol tastants such as saccharin and quinine were measured. No differences in tastant preference or total fluid intake were found between ρ1 null and wild type mice of either sex (Figures S1 and S2).

During limited access to 15% ethanol without free choice (one-bottle DID model), null mutant and wild type male and female mice consumed similar amounts of ethanol both during the first 3 days with 2-hour access, and on day 4 with 4-hour access to ethanol (Figure S3).

Over 20 days of intermittent (every other day) drinking, no significant differences in amount of ethanol consumed, preference for ethanol, or total amount of fluid consumed were found between male and female ρ1 null and wild type mice (Figure S4).

### Conditioned taste aversion

**Figure 2:**
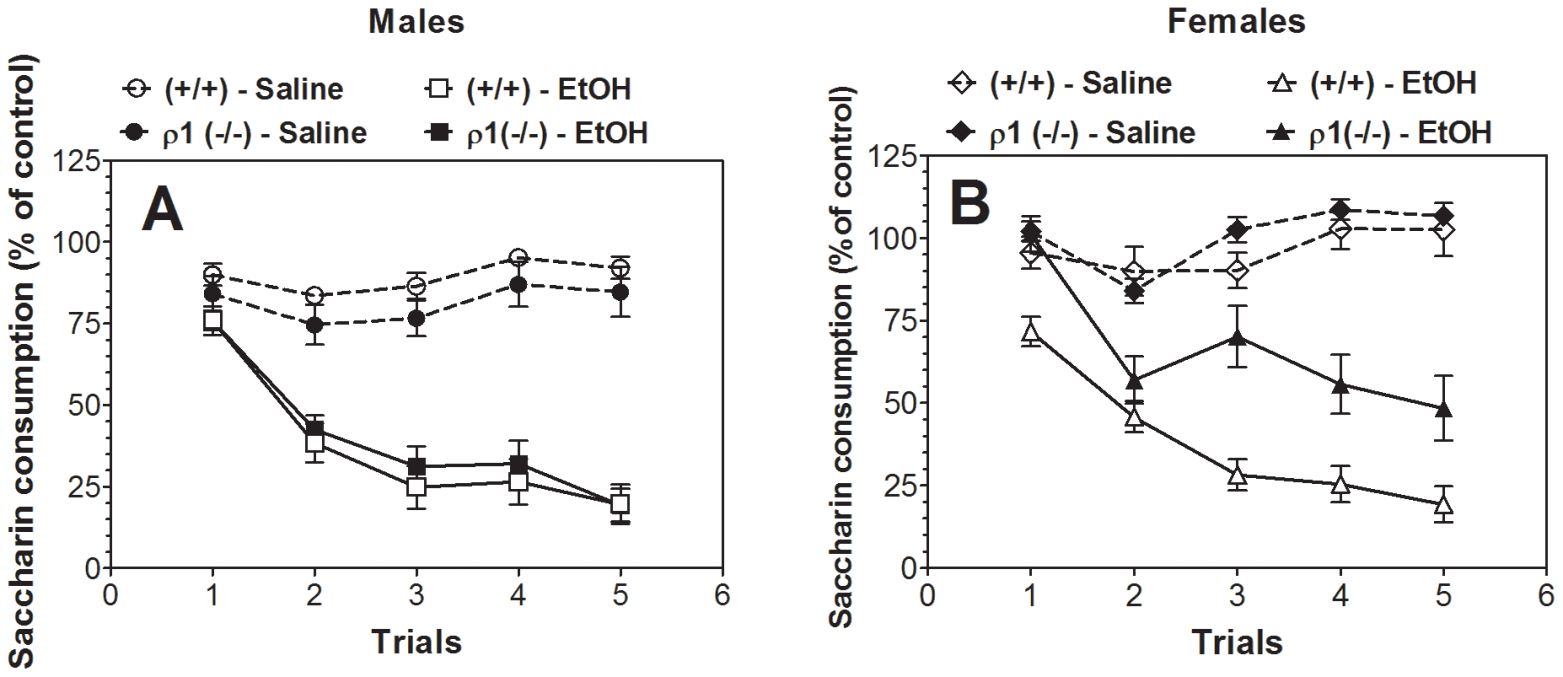
Female ρ1 (−/−) mice showed decreased ethanol conditioned taste aversion. Data represent the changes in saccharin consumption produced by injection of saline or ethanol expressed as percent of control trial (Trial 0). A. Development of CTA in males (n = 9–10 for saline injection for both genotypes; n = 20 for ethanol injection for both genotypes). Saline-Ethanol pairings for wild type mice: (F_1,28_ = 44.9, p<0.001, effect of treatment; F_4,112_ = 22.4, p<0.001, dependence on trial; F_4,112_ = 24.9, p<0.001, treatment x trial interaction). Saline-Ethanol pairings for ρ1 null mice: (F_1,27_ = 28.3, p<0.001, effect of treatment; F_4,108_ = 19.4, p<0.001, dependence on trial; F_4,108_ = 19.0, p<0.001, treatment x trial interaction). Genotype-Saline pairings: (F_4,68_ = 6.6, p<0.001, effect of trial; no dependence on genotype or genotype-trial interaction). Genotype-Ethanol pairings: (F_4,152_ = 101, p<0.001, effect of trial; no dependence on genotype or genotype-trial interaction). B. Development of CTA in females (n = 9–10 for saline injection for both genotypes; n = 12–20 for ethanol injection for both genotypes). Saline-Ethanol pairings for wild type mice: (F_1,128_ = 64.4, p<0.001, effect of treatment; F_4,112_ = 16.5, p<0.001, dependence on trial; F_4,112_ = 25.8, p<0.001, treatment x trial interaction). Saline-Ethanol pairings for ρ1 null mice: (F_1,19_ = 14.3, p<0.01, effect of treatment; F_4,76_ = 26.2, p<0.001, dependence on trial; F_4,76_ = 25.2, p<0.001, treatment x trial interaction). Genotype-Saline pairings: (F_4,68_ = 10.5, p<0.001, effect of trial; no dependence on genotype or genotype-trial interaction). Genotype-Ethanol pairings: (F1_,30_ = 12.3, p<0.001, effect of genotype, F_4,120_ = 80.9, p<0.001, dependence on trial and no dependence on genotype; F_4,120_ = 5.9, p<0.001, genotype x trial interaction). Values represent mean ± S.E.M. Data were analyzed by two-way ANOVA. ρ1 (−/−) = ρ1 null mice; (+/+) = wild type mice; EtOH =  ethanol.

There were no differences in consumption of saccharin during trial 0 (before conditioning) between wild type and null mutant mice (99.8±4 and 112±3.2 g/kg body weight for females; 92.7±3.7 and 93.9±5.6 g/kg body weight for males); however, in order to minimize initial fluctuations in tastant intake and any small differences between sexes, intake was calculated as a percentage of the trial 0 consumption for each subject by dividing the amount of saccharin solution consumed on subsequent conditioning trials by the amount of saccharin solution consumed on trial 0 (before conditioning). Ethanol-saccharin pairings reduced saccharin intake across trials compared with saline-saccharin pairings, indicating the development of CTA in both genotypes of male mice (Fig. 2A) as well as in female mice (Fig. 2B). No differences were found between saline- or ethanol-treated groups of wild type or ρ1 null male mice (Fig. 2A) or between saline-treated groups of wild type and null mutant females (Fig. 2B). However, wild type female mice developed significantly stronger CTA following ethanol treatment than null mutant females (Fig. 2B).

### Place conditioning

**Figure 3:**
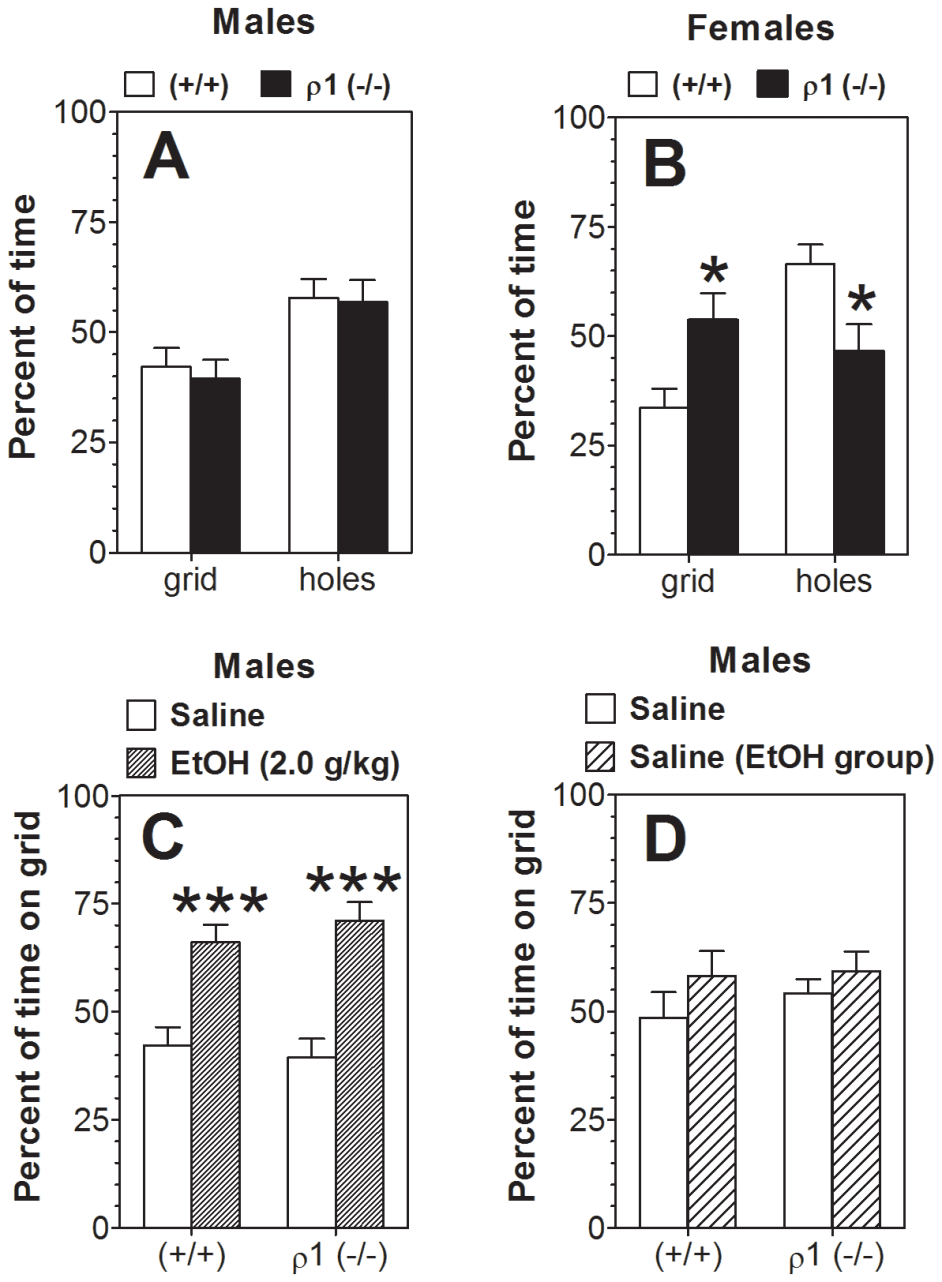
Ethanol-induced conditioned place preference in ρ1 (−/−) mice. Data represent the percent of time spent on different types of floor (A, B) or on the grid floor (C, D). A. Males (n = 13–15 per genotype; F_1,52_ = 14, p<0.001, main effect of floor). B. Females (n = 6 per genotype; F_1,20_ = 5.7, p<0.05, main effect of floor; F_1,20_ = 13.7, p<0.01, genotype x floor interaction, *p<0.05 vs. another genotype on the same type of floor). C. Males, 1^st^ Preference test (n = 13–15 per genotype and treatment; F_1,52_ = 43, p<0.001, main effect of treatment, ***p<0.001 vs. saline group of corresponding genotype). D. 2^nd^ Preference test (Extinction). Values represent mean ± S.E.M. Data were analyzed by two-way ANOVA with Bonferroni *post hoc* test. ρ1 (−/−) = ρ1 null mice; (+/+) = wild type mice; EtOH = ethanol.

Following control saline injections, male mice spent substantially less time on the grid floor than the floor with round holes (Fig. 3A). However, no significant difference between genotypes was found. Wild type female mice also spent less time on the grid floor than the floor with round holes (Fig. 3B). Post-hoc analysis showed that wild type female mice, compared with their ρ1 null littermates, spent less time on the grid floor (p<0.05) but more time on the floor with holes (p<0.05). Taking into account this preference for floor type in female mice of one genotype, only male mice were used in further experiments, and we calculated place conditioning only for the mice injected with ethanol paired with their less favorite type of floor (the grid floor). The percent of time spent on the grid floor by saline- and ethanol-injected male mice of each genotype is shown in Fig. 3C. Male mice of both genotypes spent more time on the grid floor when paired with ethanol than when paired with saline, reflecting development of conditioned place preference. However, there was no difference in development of place conditioning between the genotypes. After 6 days of extinction, there were no differences between wild type and ρ1 null male mice in time spent on the different types of floor (Fig. 3D).

### Loss of righting reflex

**Figure 4:**
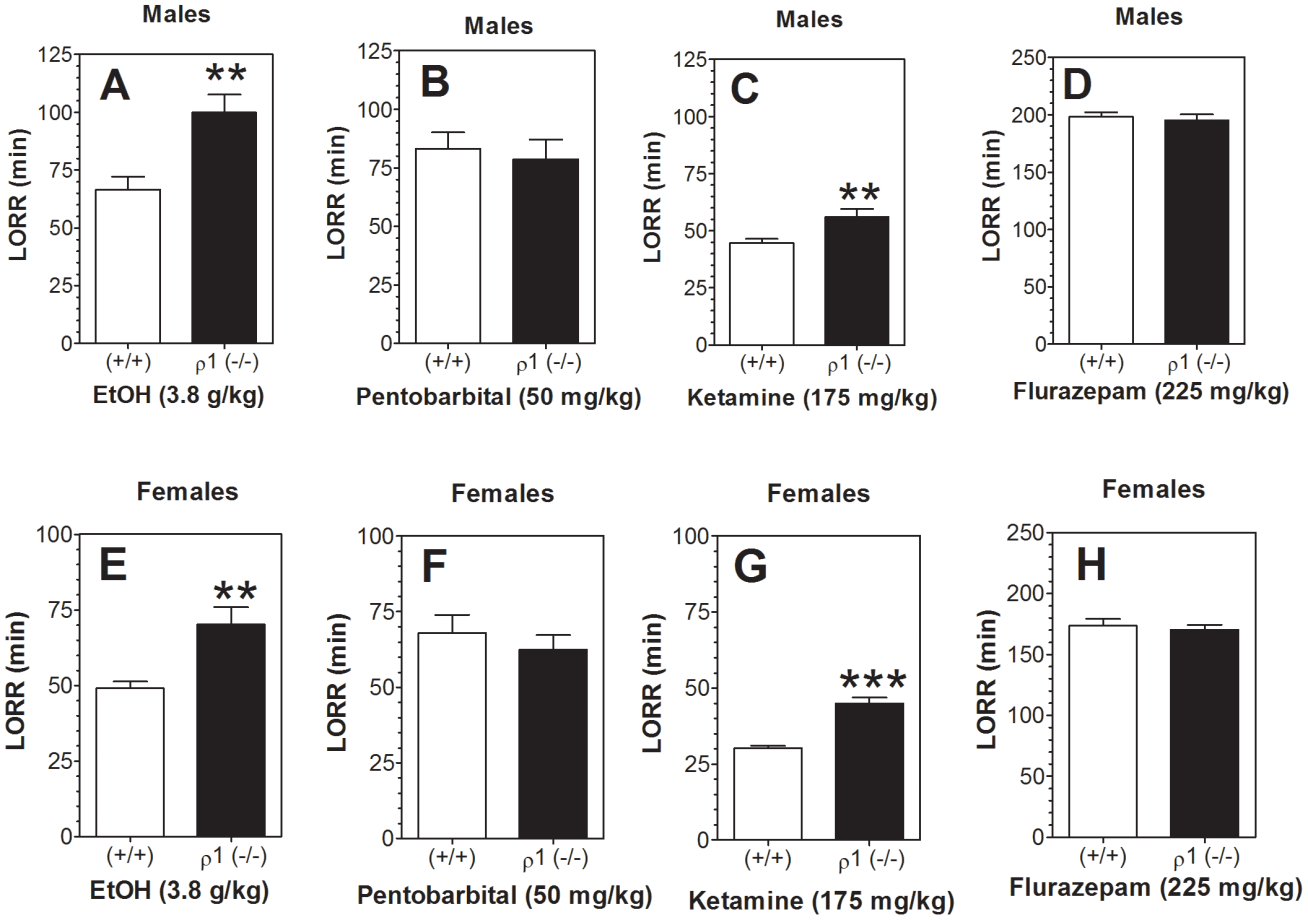
Lack of ρ1 increased duration of LORR by ethanol and ketamine but not pentobarbital or flurazepam. A, B, C, D – Males. E, F, G, H – Females. A, E – Ethanol (n = 8–10 per genotype for both sexes; t(16) = 3.3 for males and females, **p<0.01 vs. wild type of corresponding genotype). B, F – Pentobarbital (n = 12–15 per genotype for both sexes). C, G – Ketamine (n = 10–14 per genotype for both sexes; t(25) = 2.9 for males and t(22) = 6.3 for females, **p<0.01, ***p<0.001 vs. wild type of corresponding genotype). D, H – Flurazepam (n = 7–10 per genotype for both sexes). Values represent mean ± S.E.M. Data were analyzed by Student's t-test. ρ1 (−/−) = ρ1 null mice; (+/+) = wild type mice; EtOH = ethanol; LORR = loss of righting reflex.

Duration of LORR was measured in ρ1 null and wild type mice of both sexes following the injection of four sedative agents (ethanol, flurazepam, pentobarbital, or ketamine). For ethanol, there was a longer duration of LORR for ρ1 null mice (Fig. 4A and E). Similar to ethanol, ketamine also significantly prolonged the duration of LORR in ρ1 null mice (Fig. 4C and G). No differences in duration of LORR between null mutant and wild type mice were found after administration of flurazepam and pentobarbital (Fig. 4B, D, F and H). No gender-dependent differences in effects of the drugs were found.

### Acute ethanol withdrawal severity

A single 4.0 g/kg ethanol dose suppressed basal HIC in ρ1 null and wild type mice of both sexes for about 5 hours, followed by increased HIC (Figure S5A and B). Male and female ρ1 null and wild type mice did not differ in levels of basal HIC. Animals of both genotypes and sexes demonstrated signs of withdrawal (HIC scores higher than the basal level). However, there were no differences in area under the curves for HIC and above the basal level during withdrawal for either females (1.1±0.6 and 2.2±0.5 for wild type and null mutant mice, respectively) or males (1.9±0.5 and 1.6±0.4 for wild type and null mutant mice, respectively) (Figure S5C and D).

### Startle response

No differences in the acoustic startle responses were observed between wild type and ρ1 null male or female mice (Figure S6).

### Ethanol-induced motor incoordination

**Figure 5:**
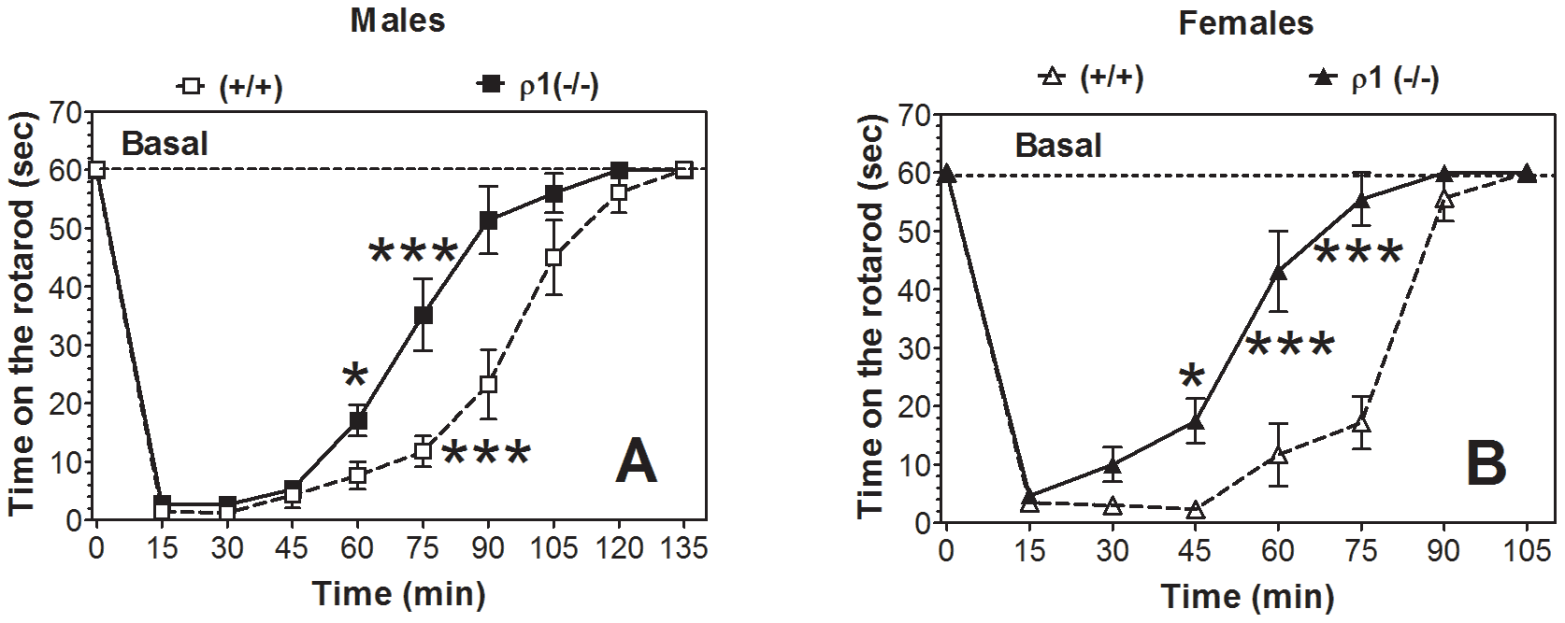
Faster recovery from motor incoordinating effect of ethanol in ρ1 (−/−) mice. Data represent time (sec) on the rotarod after injection of ethanol (2.0 g/kg). A. Males (n = 5–7 per genotype; F_1,16_ = 10, p<0.01, dependence on genotype; F_9, 144_ = 163, p<0.001, dependence on time; F_9,144_ = 6.8, p<0.001, genotype x time interaction). B. Females (n = 6–8 per genotype; F_1,12_ = 30.5, p<0.001, dependence on genotype; F_7,84_ = 124, p<0.001, dependence on time; F_7,84_ = 11.6, p<0.001, genotype x time interaction). Data represent mean ± S.E.M. Data were analyzed by two-way ANOVA with Bonferroni *post hoc* test (*p<0.05, ***p<0.001 vs. wild type genotype for each time point). ρ1 (−/−) = ρ1 null mice; (+/+) = wild type mice.

Acute administration of ethanol (2.0 g/kg) produced motor incoordination in both genotypes, but ρ1 null mice of both sexes recovered from this impairment faster than wild type mice (Fig. 5A and B).

The ability of a mouse to maintain position on the rotarod under ethanol intoxication is the result of several types of more simple *in vivo* responses, such as the anxiolytic effect of ethanol, its motor activating or sedative effects, and myorelaxation. The effects of low doses of ethanol were studied in the corresponding tests given that differences in rotarod motor-incoordination were seen for doses lower than 2.0 g/kg (recovery) but not for the initial 2.0 g/kg dose of ethanol, taking into account the high metabolism of ethanol in mice.

Ethanol at 1.0 and 1.5 g/kg did not change the number of missteps either in male or female mice of either genotype (Figure S7A and B). In contrast, ethanol injection significantly decreased the grip strength compared with saline (F_2,28_ = 18, *p*<0.001, main effect of treatment for male mice; F_2,28_ = 42, *p*<0.001, main effect of treatment for female mice) (Figure S7C and D), but no genotype-dependent differences were found.

### Anxiety-related behavior

**Figure 6:**
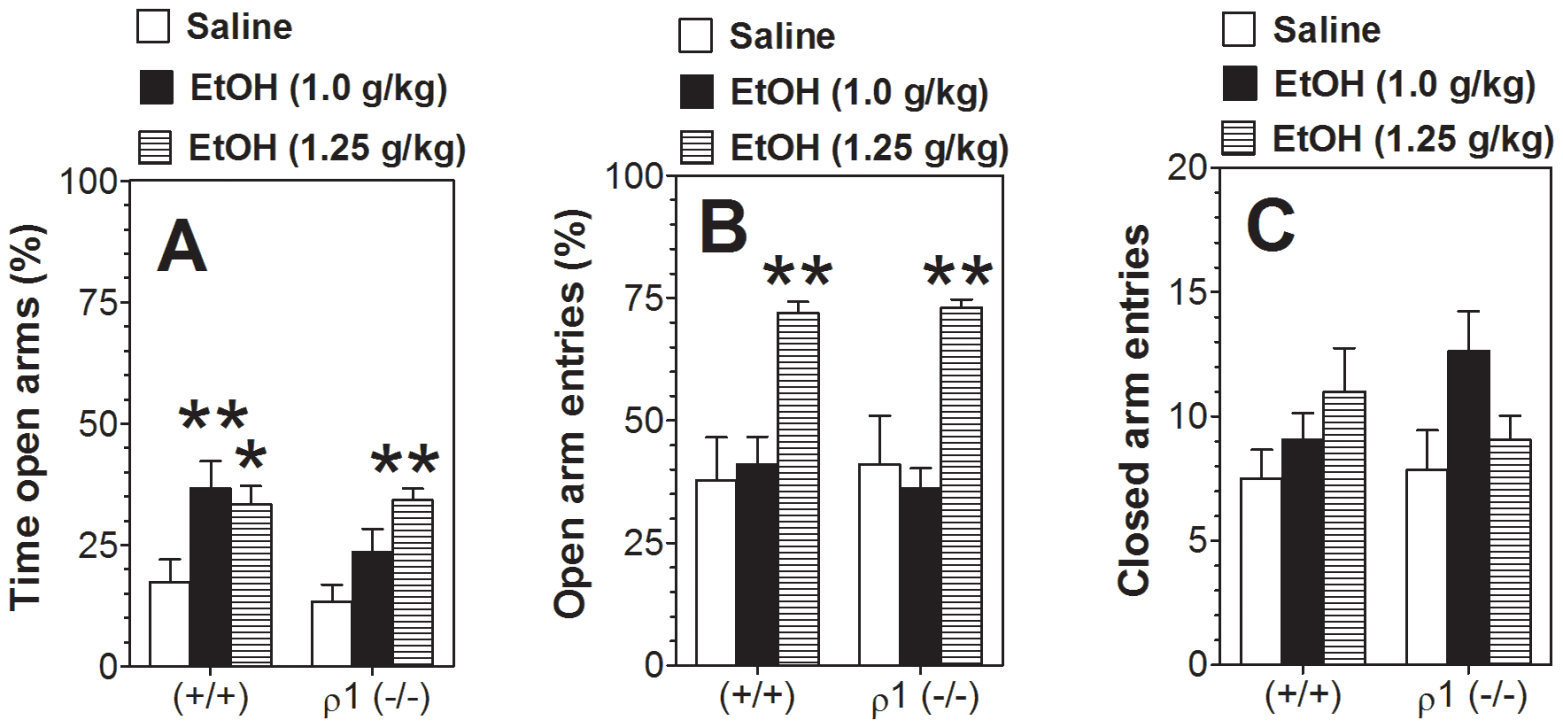
Ethanol reduced anxiety-related behavior equally in the elevated plus-maze in wild type and ρ1 null mice. A. Percent of time in open arms. B. Percent of entries into the open arms. C. Number of entries into the closed arms. Data from females and males were combined since there were no gender differences. Values represent mean ± S.E.M. Data were analyzed by two-way ANOVA with Bonferroni *post hoc* test. *p<0.05, **p<0.01 vs. saline group of corresponding genotype (n = 13–19 per group). ρ1 (−/−) = ρ1 null mice; (+/+) = wild type mice; EtOH = ethanol.

In the plus-maze, locomotor activity was assessed by number of entries into the closed arms, whereas anxiety-related behavior was measured by percentage of time spent in open arm entries after injection of saline or ethanol. Because no gender-dependent differences were found, the data from male and female mice were combined for the final analysis. Ethanol treatment affected the percentage of time spent in open arms (F_2,93_ = 12, *p*<0.001) (Fig. 6A) as well as the percentage of open arm entries (F_2,93_ = 18, *p*<0.001) (Fig. 6B). No differences between genotypes for either parameter were found. Post-hoc analysis showed that ethanol at doses of 1.0 and 1.25 g/kg significantly increased the percentage of time spent in open arms in wild type mice, whereas a significant increase in this behavior was seen only at the 1.25 g/kg dose in ρ1 null mice. The percentage of open arm entries was increased by 1.25 g/kg ethanol in both wild type and ρ1 null mice. The number of closed arm entries was not dependent on genotype or treatment (Fig. 6C).

### Spontaneous locomotion

**Figure 7:**
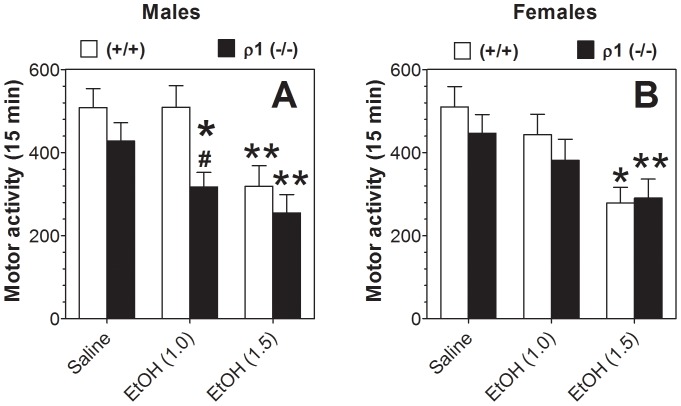
Effect of ethanol on motor activity after pre-habituation. A. Males (n = 12–20 per genotype; F_1,30_ = 4.8, p<0.05 main effect of genotype; F_2,60_ = 13.8, p<0.001 main effect of treatment; no genotype x treatment interaction). B. Females (n = 13–18 per genotype; F_2,56_ = 18.4, p<0.001 main effect of treatment; no dependence on genotype or genotype x treatment interaction). Values represent mean ± S.E.M. Data were analyzed by two-way ANOVA with repeated measures with Bonferroni *post hoc* test (*^#^*p<0.05 vs. response of another genotype for the same condition). Effect of ethanol within each genotype was also analyzed by one-way ANOVA with repeated measures with Dunnett's *post hoc* test (*p<0.05, **p<0.01 vs. saline response of corresponding genotype). ρ1 (−/−) = ρ1 null mice; (+/+) = wild type mice; EtOH = ethanol.

We studied effects of ethanol on motor activity after habituation to the experimental cage and control saline injection. No differences in baseline (saline injection) motor activity were found between wild type and ρ1 null mice of either sex. Ethanol dose-dependently reduced motor activity for all mice (Fig. 7A and B). However, male null mutant mice were more sensitive to sedation induced by 1.0 g/kg of ethanol than wild type littermates (Fig. 7A), while female wild type and null mutant mice did not show any effect at this concentration (Fig. 7B). One-way ANOVA within each genotype showed that 1.5 g/kg ethanol significantly reduced motor activity in both genotypes and sexes (Fig. 7A and B).

### Pharmacological replication of *in vivo* effects in ρ1 null mutant mice

To determine whether the different effects of ethanol observed in the null mutant mice were the result of deletion of ρ1 or compensatory changes resulting from loss of the subunit, we studied the effects of two mixed ρ1/ρ2 antagonists - (S)-ACPBPA with higher selectivity for ρ1 subunit (“ρ1” selective antagonist) and (R)-ACPBPA with higher selectivity for ρ2 subunit (“ρ2” selective antagonist) ([41] and personal communication). For these experiments, we chose three tests showing the most prominent differences between ρ1 null and wild type mice.

**Figure 8:**
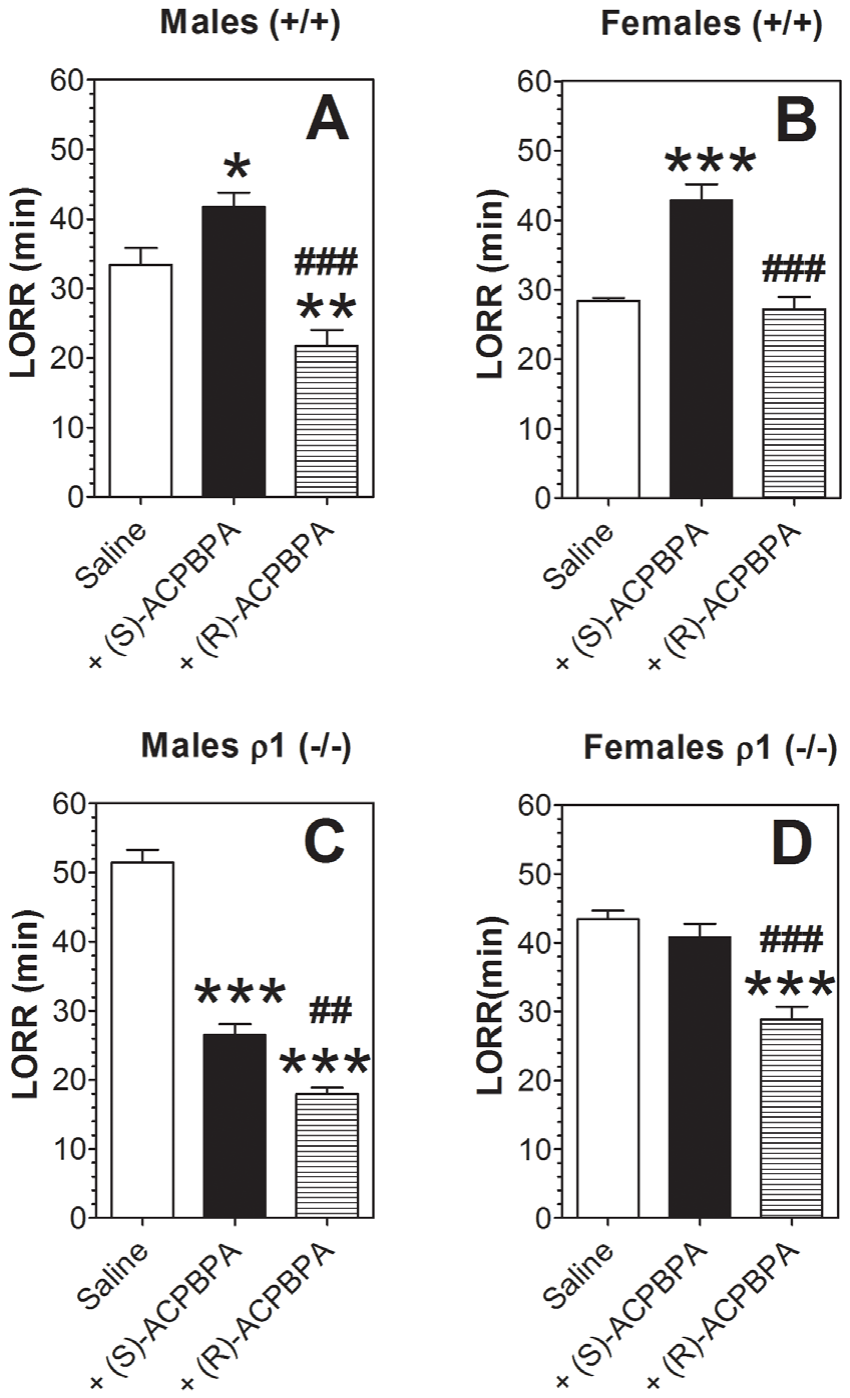
Effect of ρ1/ρ2 antagonists on ethanol (3.4 g/kg)-induced LORR in wild type and ρ1 (−/−) mice. A. Wild type male mice. (n = 9–11; F_2,27_ = 17.9, p<0.001). B. Wild type female mice. (n = 9–10; F_2,26_ = 25, p<0.001). C. ρ1 (−/−) male mice. (n = 7–11; F_2,25_ = 113, p<0.001). D. ρ1 (−/−) female mice. (n = 9–10; F_2,27_ = 20.7, p<0.001). *p<0.05, **p<0.01, ***p<0.001 vs. saline; ^##^p<0.01, ^###^p<0.001 (S)-ACPBPA vs. (R)-ACPBPA). Values represent mean ± S.E.M. Data were analyzed by one-way ANOVA with Bonferroni *post hoc* test. ρ1 (−/−) = ρ1 null mice; (+/+) = wild type mice; LORR = loss of righting reflex.

In wild type mice of both sexes, the “ρ1” antagonist increased duration of LORR induced by ethanol (Fig. 8A and B). In contrast, the “ρ2” antagonist did not change the duration of ethanol-induced LORR in wild type female mice and reduced it in wild type males. In ρ1 null mice, the “ρ1” antagonist reduced the duration of ethanol-induced LORR in males (Fig. 8C) but did not change it in females (Fig. 8D). In contrast, the “ρ2” antagonist reduced the duration of LORR in null mutant mice of both sexes. It should be noted that in ρ1 null mice, the reduction of duration of ethanol-induced LORR by the “ρ2” antagonist was greater than the effect of the “ρ1” antagonist.

**Figure 9:**
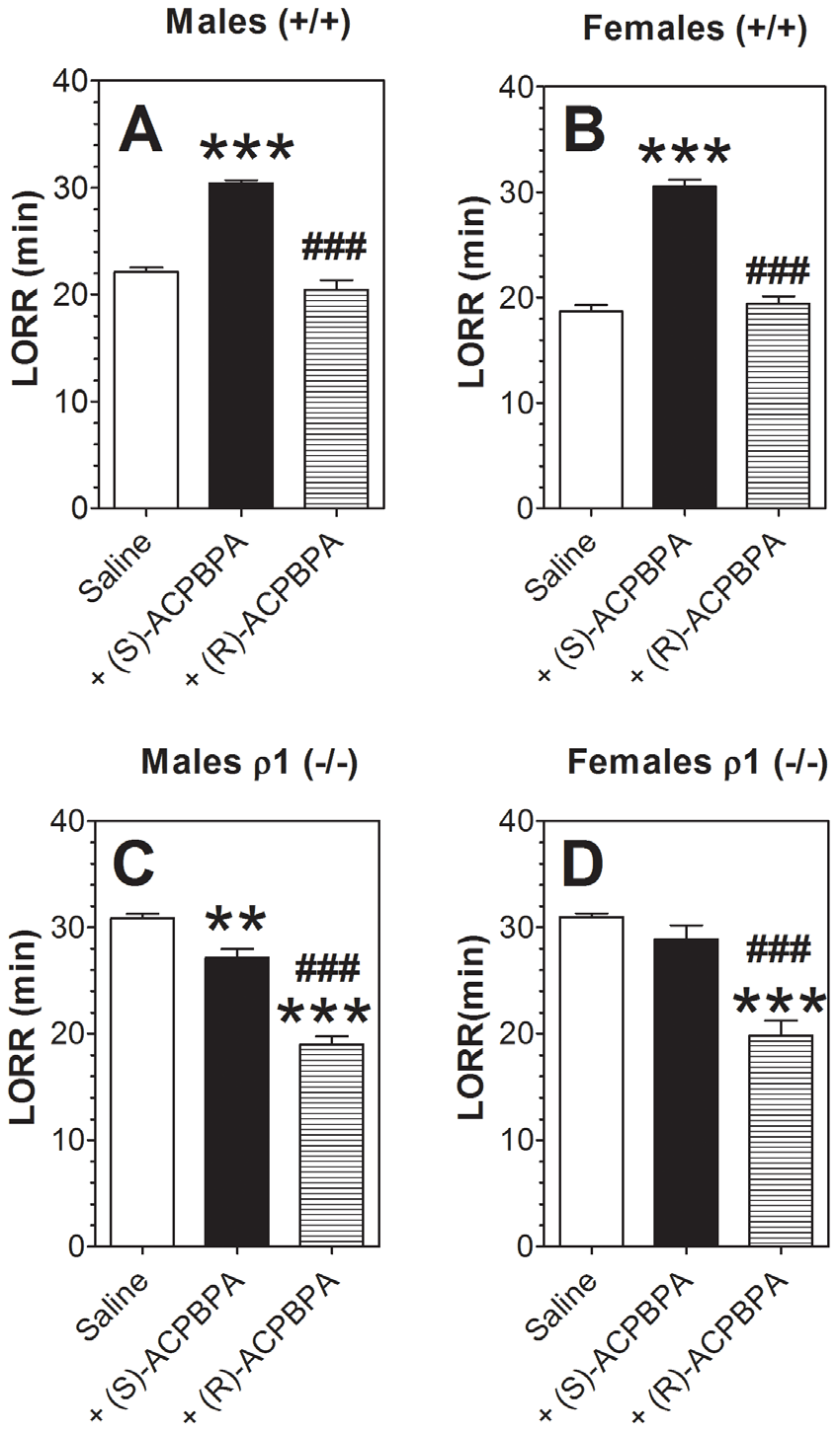
Effect of ρ1/ρ2 antagonists on ketamine (150 mg/kg)-induced LORR in wild type and ρ1 (−/−) mice. A. Wild type male mice. (n = 9–11; F_2,12_ = 77.5, p<0.001). B. Wild type female mice. (n = 9–10; F_2,12_ = 105, p<0.001). C. ρ1 (−/−) male mice. (n = 7–11; F_2,12_ = 79.3, p<0.001). D. ρ1 (−/−) female mice. (n = 9–10; F_2,12_ = 27.7, p<0.001). **p<0.01, ***p<0.001 vs. saline; ^###^p<0.001 (S)-ACPBPA vs. (R)-ACPBPA). Values represent mean ± S.E.M. Data were analyzed by one-way ANOVA with Bonferroni *post hoc* test. ρ1 (−/−) = ρ1 null mice; (+/+) = wild type mice; LORR = loss of righting reflex.

Very similar effects of the “ρ1/ρ2” drugs were seen on duration of LORR induced by ketamine. In wild type mice of both sexes, the “ρ1” antagonist increased duration of LORR induced by ketamine (Fig. 9 A and B). In contrast, the “ρ2” antagonist did not change the duration of ketamine-induced LORR in wild type mice of either sex. In ρ1 null mice, the “ρ1” antagonist slightly reduced the duration of ketamine-induced LORR in male mice (Fig. 9C) but did not change it in females (Fig. 9D). In contrast, the “ρ2” antagonist significantly reduced the duration of LORR in null mutant mice of both sexes. As was seen for ethanol in ρ1 null mice, the reduction in duration of ketamine-induced LORR was greater for the “ρ2” than for the “ρ1” antagonist.

**Figure 10:**
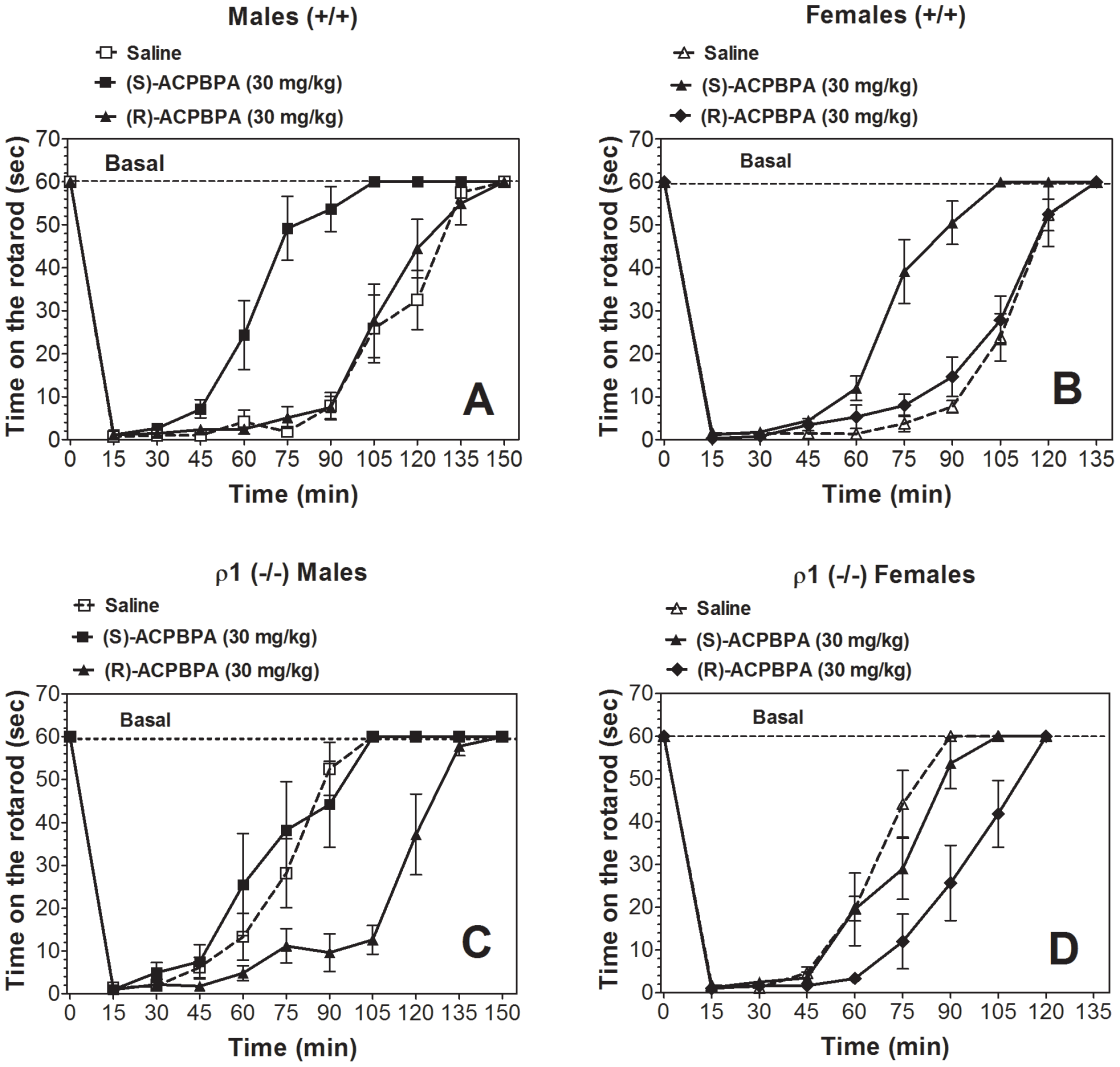
Effect of ρ1/ρ2 antagonists on recovery from ethanol (2.0 g/kg)-induced motor incoordination in wild type and ρ1 (−/−) mice. A. Wild type male mice, n = 6. (S)-ACPBPA (F_1,10_ = 43.3, p<0.001, effect of treatment; F_10,100_ = 107, p<0.001, effect of time; F_10,100_ = 17.1, p<0.001, treatment x time interaction). (R)-ACPBPA (F_10,100_ = 120, p<0.001, effect of time; no effect of treatment or treatment x time interaction). B. Wild type female mice, n = 6. (S)-ACPBPA (F_1,10_ = 69, p<0.001, effect of treatment; F_9,90_ = 196, p<0.001, effect of time; F_9,90_ = 24, p<0.001, treatment x time interaction). (R)-ACPBPA (F_9,90_ = 181, p<0.001, effect of time; no effect of treatment or treatment x time interaction). C. ρ1 (−/−) male mice, n = 4–6. (S)-ACPBPA (F_10,80_ = 67.7, p<0.001, effect of time; no effect of treatment or treatment x time interaction). (R)-ACPBPA (F_1,9_ = 31.5, p<0.001, effect of treatment; F_10,90_ = 102, p<0.001, effect of time; F_10,90_ = 13.4, p<0.001, treatment x time interaction). D. ρ1 (−/−) female mice, n = 6. (S)-ACPBPA (F_8,80_ = 126, p<0.001, effect of time; no effect of treatment or treatment x time interaction). (R)-ACPBPA (F_1,10_ = 24.1, p<0.001, effect of treatment; F_8,80_ = 101, p<0.001, effect of time; F_8,80_ = 8.1, p<0.001, treatment x time interaction). Values represent mean ± S.E.M. Data were analyzed by two-way ANOVA with repeated measures with Bonferroni *post hoc* test vs. corresponding saline-injected mice. ρ1 (−/−) = ρ1 null mice; (+/+) = wild type mice.

Similar effects of the antagonists were also observed in the recovery from acute ethanol-induced motor incoordination. In wild type mice of both sexes the “ρ1” antagonist accelerated the recovery (Fig.10A and B). In contrast, the “ρ2” antagonist did not change the recovery from ethanol-induced motor incoordination in wild type mice. In ρ1 null mice of both sexes, the “ρ1” antagonist did not change recovery from the motor incoordination effect of ethanol (Fig.10C and D). However, the “ρ2” antagonist significantly slowed the motor recovery in null mutant mice of both sexes.

### Ethanol action on recombinant receptors

Overall, the pharmacological analyses showed that administration of the “ρ1” antagonist to wild type mice mimicked the *in vivo* changes by ethanol observed in ρ1 null mice but did not produce these effects in mice lacking ρ1 (with one exception). In contrast, the “ρ2” antagonist did not change ethanol action in wild type mice but produced *in vivo* effects in mice lacking ρ1 that were opposite of the effects from deleting (or inhibiting) ρ1. These results indicate that a role for ρ2 in regulation of ethanol responses may be revealed when ρ1 is deleted. Receptors formed from ρ1 are inhibited by low concentrations of ethanol [17], in contrast to other GABA_A_ receptors containing α, β, γ, and δ subunits, which are potentiated by ethanol. The opposite effects of the “ρ1” and “ρ2” antagonists in the ρ1 null mice raise the question of whether ethanol inhibits or enhances function of GABA_A_ receptors formed from the ρ2 subunit.

**Figure 11:**
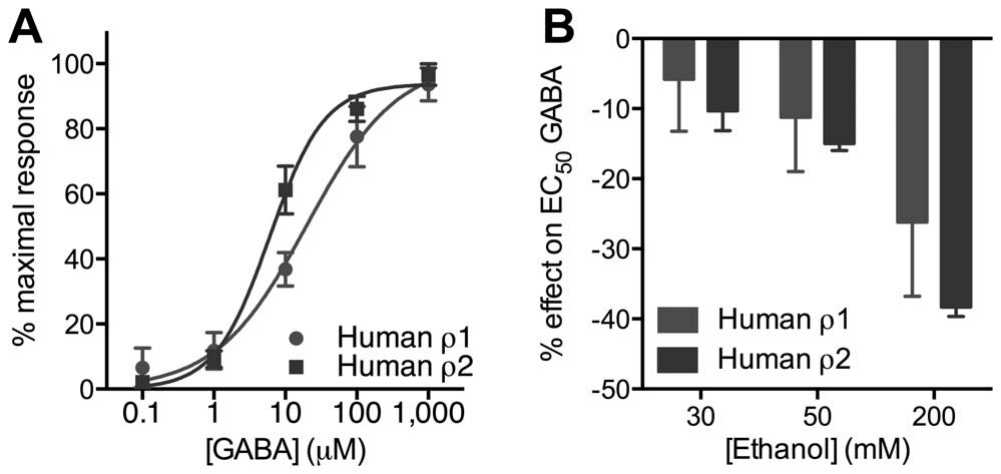
GABA sensitivity and ethanol modulation of currents produced by human ρ1 or ρ2 recombinant receptors in oocytes. A. GABA concentration-response curve for ρ1 (n = 5) and ρ2 (n = 6) GABA_A_ receptors. B. Ethanol modulation of EC_50_ GABA-mediated currents for ρ1 (n = 4–5) and ρ2 (n = 3–9) GABA_A_ receptors (no significant difference, two-way ANOVA).

We found that the sensitivity to GABA was similar for both ρ1- and ρ2-containing receptors expressed in a heterologous system. From the concentration-response curves (Fig. 11A), we determined the GABA EC_50_ values. The GABA EC_50_ values were 20.1 (6.59 to 61.3) for ρ1 and 6.05 (4.19 to 8.74) for ρ2 (95% confidence intervals). There were no differences in the ethanol modulation between ρ1 and ρ2. Increasing concentrations of ethanol (30–200 mM) dose-dependently inhibited the EC_50_ GABA-mediated current (Fig. 11B).

### Measurement of GABA_A_ receptor subunit mRNAs

Because our data show gender-dependent differences in several ethanol-induced effects *in vivo*, we compared the expression of GABA_A_ receptor subunits (ρ1, ρ2, α2, α6) in wild type and ρ1 null mice. The results confirm an absence of ρ1 and show that ρ2, α2, and α6 mRNA expression did not differ between wild type and ρ1 null mice or between males and females. Combining males and females, the ratios of mRNA levels (null/wild type were 1.12, 0.86, and 0.98 for ρ2, α2, and α6, respectively (none show a statistical difference from 1.0). It is worth noting that ρ1 had an average Cq value of 31, whereas the other target genes' average Cq values ranged between 19 and 27, clearly showing that ρ1 is expressed at a lower level than the other GABA_A_ mRNAs studied.

### Ethanol metabolism

There were no differences in metabolism of ethanol (4.0 g/kg dose) between wild type and ρ1 null mice in either sex (data not shown). The slopes of the regression lines were −49.4±2.0 (wild type males, n = 6), −47.8±3.4 (null mutant males, n = 6), −62.1±3.0 (wild type females, n = 6), and -70.4±3.0 (null mutant females, n = 6).

## Discussion

Deletion of ρ1 alters multiple ethanol-induced effects *in vivo* (for summary of phenotypes, see Table 1). The major *in vivo* changes produced by ρ1 deletion were increased sedative (hypnotic) effects of ethanol and acceleration of recovery from acute ethanol-induced motor incoordination. Other changes, which were gender-specific, include reduced ethanol intake and preference in male ρ1 null mice and reduced development of ethanol-induced conditioned taste aversion in female ρ1 null mice. Gender-specific effects of gene deletion are common in studies of ethanol effects [37]. One potential problem in interpretation of results obtained with global knockout mice is compensatory changes in expression of other genes as a result of deletion of the target gene [37], [42]. In this context, it is important to note that two major *in vivo* differences between wild type and null mutant mice, recovery from acute ethanol intoxication and sedative (LORR) effects of ethanol, were reproduced in wild type mice after administration of the “ρ1” antagonist. The “ρ2” antagonist did not affect wild type mice, except for a slight reduction of LORR in males. In mice lacking ρ1, the “ρ2” antagonist reduced the intoxicating and sedative effects of ethanol to approximately the level of ethanol responses of wild type mice. These results suggest a functional interaction between ρ1 and ρ2 subunits with a dominant role of ρ1 since the role of ρ2 was revealed only when ρ1 was absent. Similarity between ρ1 and ρ2 subunits is also supported by our data showing that ethanol inhibits the function of both homomeric ρ2 and ρ1 GABA_A_ receptors.

**Table 1:** Summary of *in vivo* effects of ethanol in mice lacking ρ1 subunit of GABA_A_ receptors.

Test	*In Vivo* Response	Drug	Dose/Concentrations	Males	Females
2 Bottle Choice	Intake (g/kg/24 hrs)	EtOH	3–18%	**↓**	=
	Preference	EtOH	3–18%	**↓**	=
	Fluid Intake (g/kg/24 hrs)	EtOH	3–18%	=	=
2 Bottle choice	Preference	Saccharin	0.165–0.66%	=	=
	Fluid Intake (g/kg/24 hrs)	Saccharin	0.165–0.66%	=	=
2 Bottle choice	Preference	Quinine	0.03–0.06 mM	=	=
	Fluid Intake (g/kg/24 hrs)	Quinine	0.03–0.06 mM	=	=
2 Bottle choice –intermittent	Intake (g/kg/24 hrs)	EtOH	15%	=	=
	Preference	EtOH	15%	=	=
	Fluid Intake (g/kg/24 hrs)	EtOH	15%	=	=
1 bottle – DID	Intake (g/kg/2-4 hrs)	EtOH	15%	=	=
LORR	Duration	EtOH	3.8 g/kg	**↑**	**↑**
		Pentobarbital	50 mg/kg	=	=
		Ketamine	175 mg/kg	**↑**	**↑**
		Flurazepam	225 mg/kg	=	=
Rotarod	Recovery	EtOH	2.0 g/kg	**←**	**←**
Startle reflex				=	=
Acute withdrawal		EtOH	4.0 g/kg	=	=
CTA		EtOH	2.5 g/kg	=	**↓**
CPP		EtOH	2.0 g/kg	=	NA
Elevated Plus Maze	Anxiety-like behavior	EtOH	1.0 g/kg	=	=
			1.25 g/kg	=	=
Motor activity		EtOH	1.0 g/kg	↑	=
			1.5 g/kg	=	=
Grip strength		EtOH	1.0 g/kg	=	=
			1.5 g/kg	=	=
Missteps		EtOH	1.0 g/kg	=	=
			1.5 g/kg	=	=
Metabolism		EtOH	4.0 g/kg	=	=

CTA = conditioned taste aversion; CPP = conditioned place preference; LORR = loss of righting reflex; DID = drinking in the dark; EtOH = ethanol. ↓ - reduction of response in null mutant compared with corresponding wild type mice; ↑ - increase in response in null mutant compared with corresponding wild type mice;  =  - no difference between null mutant and wild type mice; ← - left shift in null mutant mice.

Rho subunits are found in many brain regions and have been characterized in the striatum, where they are found in aspiny and medium spiny neurons and astrocytes, and may contribute to synaptic and extrasynaptic GABA responses as well as gliotransmission [43], [44]. They have also been characterized biochemically and electrophysiologically in superior colliculus, hippocampus, amygdala, visual cortex, cerebellar astrocytes, and Purkinje cells [45]–[49]. Their sensitivity to low (high nanomolar-low micromolar) concentrations of GABA and their prolonged conductance due to little or no desensitization is uniquely suited to tonic, extrasynaptic inhibition [43]. Detailed evidence for GABA_A_ ρ receptor expression and functional responses in the CNS can be found in the review by Martinez-Delgado et al., 2010, where these receptors have been associated with mediating neuronal excitability in the superior colliculus, phasic inhibition at interneuron Purkinje-cell synapses, and protection against neurotoxicity in hippocampal cultures [43]. GABA_A_ ρ receptors may play a role in fear, anxiety, learning, and memory since ρ1/ρ2 antagonists enhance anxiety-related behavior in the elevated plus maze and enhance learning and memory in the Morris water maze [50], [51]. In addition to our *in vivo* data, several lines of evidence link these receptors to ethanol action: 1) ethanol inhibits the function of both ρ1 and ρ2 GABA_A_ receptors similarly; 2) there is genetic correlation of ρ1 mRNA expression with ethanol consumption and motor activation in NAc in BxD RI mice (r = 0.77, 10% ethanol preference in two-bottle choice test and r = −0.48, ethanol-induced motor response, distance traveled 0–5 minute time interval, from genenetwork.org); 3) family-based association analyses demonstrate that single nucleotide polymorphisms in both human genes (*GABRR1* and *GABRR2*) were significantly associated with alcohol dependence, and the association is strongest when the analysis is focused upon those with earlier onset of alcohol dependence [18].

As noted above, there is some evidence for co-assembly of GABA_A_ ρ receptors in the spinal cord and brain stem with other GABA_A_ subunits [11], [13] to form functional GABA_A_ and GABA_A_ ρ heteromeric receptors [11], [12]. However, lack of ρ1 does not change the duration of LORR induced by GABA_A_ receptor allosteric modulators such as flurazepam or pentobarbital. On the other hand, deletion of the ρ1 subunit is accompanied by an increase in duration of ketamine-induced LORR. Furthermore, this effect was reproduced in wild type mice by administration of a ρ1 selective antagonist and therefore was not a result of potential developmental compensation. Ketamine is an antagonist of NMDA receptor function [52]. However, it is not clear if the depressant effect of high doses of ketamine that produce LORR is mediated solely by NMDA receptor inhibition, because pharmacologically relevant concentrations also inhibit nAChRs [53], [54] and enhance GABA_A_ receptor function specifically through α6-containing GABA_A_ receptors [55]. However, we did not find any differences in expression of GABA_A_ α6-subunit in cerebella of mice lacking ρ1.

Glycine, taurine, and β-alanine may activate ρ1-containing GABA_A_ receptors at concentrations that may be reached in the synapse [14]–[16]. In addition, Pan et al. (2000) showed that the ρ1 subunit forms heteromeric receptors with glycine α1 or α2 subunits *in vitro* [56]. These findings indicate a possible interaction between glycine and ρ1-containing GABA_A_ receptors in at least some areas such as brain stem and spinal cord. Impairment of function of glycine receptors containing α1 subunits increases acoustic startle response [27], but lack of ρ1 had no effect on the acoustic startle response.

GABA_A_ receptors formed from ρ1 [17] or ρ2 subunits (this study) are characterized by a unique inhibitory response to ethanol. GABA_A_ receptors formed by other subunits (α, β, γ, and δ) are enhanced by ethanol [4]. Therefore, it is interesting to ask which responses to ethanol are changed in opposite directions after genetic deletion of ρ1 compared with deletion of other GABA_A_ subunits. Two responses consistent with this requirement are acute sedation (LORR) induced by high doses of ethanol and sedative motor responses induced by low doses of ethanol. Duration of LORR either decreased or was not changed after genetic deletion of α1, α2, β2 or δ subunits [5], [19], [57], whereas deletion of the ρ1 subunit increased the duration of LORR. Deletion of α1, α2, and α3 reduced sensitivity to ethanol-induced sedation (or increased motor activation) [1], [19], whereas deletion of ρ1 increased the sedative motor effects of ethanol (in males, table 1).

In summary, we provide the first evidence that the ρ1 subunit of GABA_A_ receptors is important for specific *in vivo* effects of ethanol. Moreover, our results suggest a role for ρ2 subunits in regulation of ethanol-induced responses. In this context it will be important to explore ethanol-induced effects in mice lacking the ρ*2* subunit, and these experiments are underway in our laboratory. Ultimately, GABA_A_ ρ receptors may play a role in several *in vivo* effects, including ethanol intake, that are relevant for alcoholism and may explain the association of polymorphisms linked with human *GABRR1* and *GABRR2* genes and alcohol dependence.

## Supporting Information

Figure S1 **Voluntary saccharin consumption was not different between ρ1 (−/−) and wild type mice in two-bottle choice paradigm.** A. Preference for saccharin in males. (F_2,36_ = 22, p<0.001, main effect of concentration; no main effect of genotype or genotype x concentration interaction). B. Preference for saccharin in females. (F_2,32_ = 34.1, p<0.001, main effect of concentration; no main effect of genotype or genotype x concentration interaction). C. Total fluid intake in males. (F_2,36_ = 7.8, p<0.01, main effect of genotype; F_2,36_ = 7.8, p<0.01, genotype x concentration interaction; no main effect of genotype). *p<0.05 vs. corresponding wild type mice for the same concentration of saccharin. D. Total fluid intake in females. (F_2,32_ = 8.6, p<0.01, main effect of concentration; no main effect of genotype or genotype x concentration interaction). Values represent mean ± S.E.M. Data were analyzed by two-way ANOVA with repeated measures with Bonferroni *post hoc* test (n = 8–10 per genotype for both sexes). ρ1 (−/−) = ρ1 null mice; (+/+) = wild type mice.
(TIFF)

Figure S2 **Voluntary quinine consumption was not different for ρ1 (−/−) and wild type mice in two-bottle choice paradigm.** A. Preference for quinine in males. (F_1,18_ = 47.4, p<0.001, main effect of concentration; no main effect of genotype or genotype x concentration interaction). B. Preference for quinine in females. (F_1,17_ = 70.7, p<0.001, main effect of concentration; no main effect of genotype or genotype x concentration interaction). C. Total fluid intake in males. No main effect of genotype, concentration or genotype x concentration interaction. D. Total fluid intake in females. (F_1,17_ = 16, p<0.001, main effect of concentration; no main effect of genotype or genotype x concentration interaction). Values represent mean ± S.E.M. Data were analyzed by two-way ANOVA with repeated measures with Bonferroni *post hoc* test (n = 8–10 per genotype for both sexes). ρ1 (−/−) = ρ1 null mice; (+/+) = wild type mice.
(TIFF)

Figure S3 **Ethanol intake in a limited access (one bottle DID) model was not different between ρ1 (−/−) and wild type mice.** The amount of ethanol consumed (g/kg) with either 2- or 4-hour access periods is shown. A. Male mice (n = 8–11 per genotype). B. Female mice (n = 7–9 per genotype). No main effect of genotype, concentration or genotype x concentration interaction for the 2-hour access period; no difference in ethanol intake between the two genotypes for the 4-hour access period for either male or female mice (Student's t-test). Values represent mean ± S.E.M. ρ1 (−/−) = ρ1 null mice; (+/+) = wild type mice; EtOH = ethanol.
(TIFF)

Figure S4 Ethanol intake in a two-bottle choice test with intermittent access to ethanol (every other day drinking) was not different between ρ1 (−/−) and wild type mice. A. Ethanol consumed (g/kg/24 hr) in males. (F_4,80_ = 3.2, p<0.05, main effect of time). B. Ethanol consumed (g/kg/24 hr) in females. (F_4,132_ = 8.5, p<0.001 main effect of concentration). C. Preference for ethanol in males. (F_4,80_ = 4.1, p<0.01, main effect of concentration). D. Preference for ethanol in females. (F_4,132_ = 14.6, p<0.001, main effect of concentration). E. Total fluid intake (g/kg/24 hr) in males. (F_4,80_ = 2.8, p<0.05, main effect of concentration). F. Total fluid intake (g/kg/24 hr) in females. (F_4,132_ = 13.4, p<0.001, main effect of concentration). No main effect of concentration or genotype x concentration interaction was found for any of the groups. Values represent mean ± S.E.M. Data were analyzed by two-way ANOVA with repeated measures with Bonferroni *post hoc* test (n = 9–10 per genotype for both sexes). ρ1 (−/−) = ρ1 null mice; (+/+) = wild type mice; EtOH = ethanol.
(TIFF)

Figure S5 **Severity of acute ethanol-induced withdrawal was not different between ρ1 (−/−) and wild type mice.** A. Males, HIC score. B. Females, HIC score. C. Males, Area under the HIC score and above the basal level. D. Females, Area under the HIC score and above the basal level. No differences between the two genotypes were found for either male or female mice (Student's t-test). Values represent mean ± S.E.M. (n = 7–10 for male and n = 9–10 for female mice of both genotypes). ρ1 (−/−) = ρ1 null mice; (+/+) = wild type mice; HIC = handling induced convulsions.
(TIFF)

Figure S6 **Acoustic startle response is not changed in ρ1 (−/−) mice of either sex.** Data represent the maximum startle amplitude (Vmax) as a function of sound intensity (decibels). A. Males (n = 9–10 per genotype; F_4,68_ = 50; p<0.001, main effect of sound intensity). B. Females (n = 12–19 per genotype; F_4,116_ = 79.6; p<0.001, main effect of sound intensity). No main effect of genotype or genotype x sound intensity interaction was found for either male or female mice. Values represent mean ± S.E.M. Data were analyzed by two-way ANOVA with repeated measures with Bonferroni *post hoc* test. ρ1 (−/−) = ρ1 null mice; (+/+) = wild type mice.
(TIFF)

Figure S7 **Ethanol produced similar alteration in grip strength and number of missteps in wild type and ρ1 (−/−) mice.** A. Number of missteps in males (n = 7–9 per genotype). B. Number of missteps in females (n = 7–9 per genotype). No dependence on genotype, dose or genotype x dose interaction was found for either male or female mice. C. Grip strength in males (n = 7–9 per genotype; F_2,28_ = 18.1; p<0.001, dependence on dose). D. Grip strength in females (n = 7–9 per genotype; F_2,28_ = 42; p<0.001, dependence on dose). No dependence on genotype or genotype x dose interaction was found for either male or female mice. Values represent mean ± S.E.M. Data were analyzed by two-way ANOVA with repeated measures with Bonferroni *post hoc* test. ρ1 (−/−) = ρ1 null mice; (+/+) = wild type mice; EtOH = ethanol.
(TIFF)
