# Supplementary figures and images for: GABAA Receptors Containing ρ1 Subunits Contribute to In Vivo Effects of Ethanol in Mice
Source: PLoS One. 2014 Jan 16;9(1):e85525. doi: 10.1371/journal.pone.0085525 (PMC3894180; doi:10.1371/journal.pone.0085525)

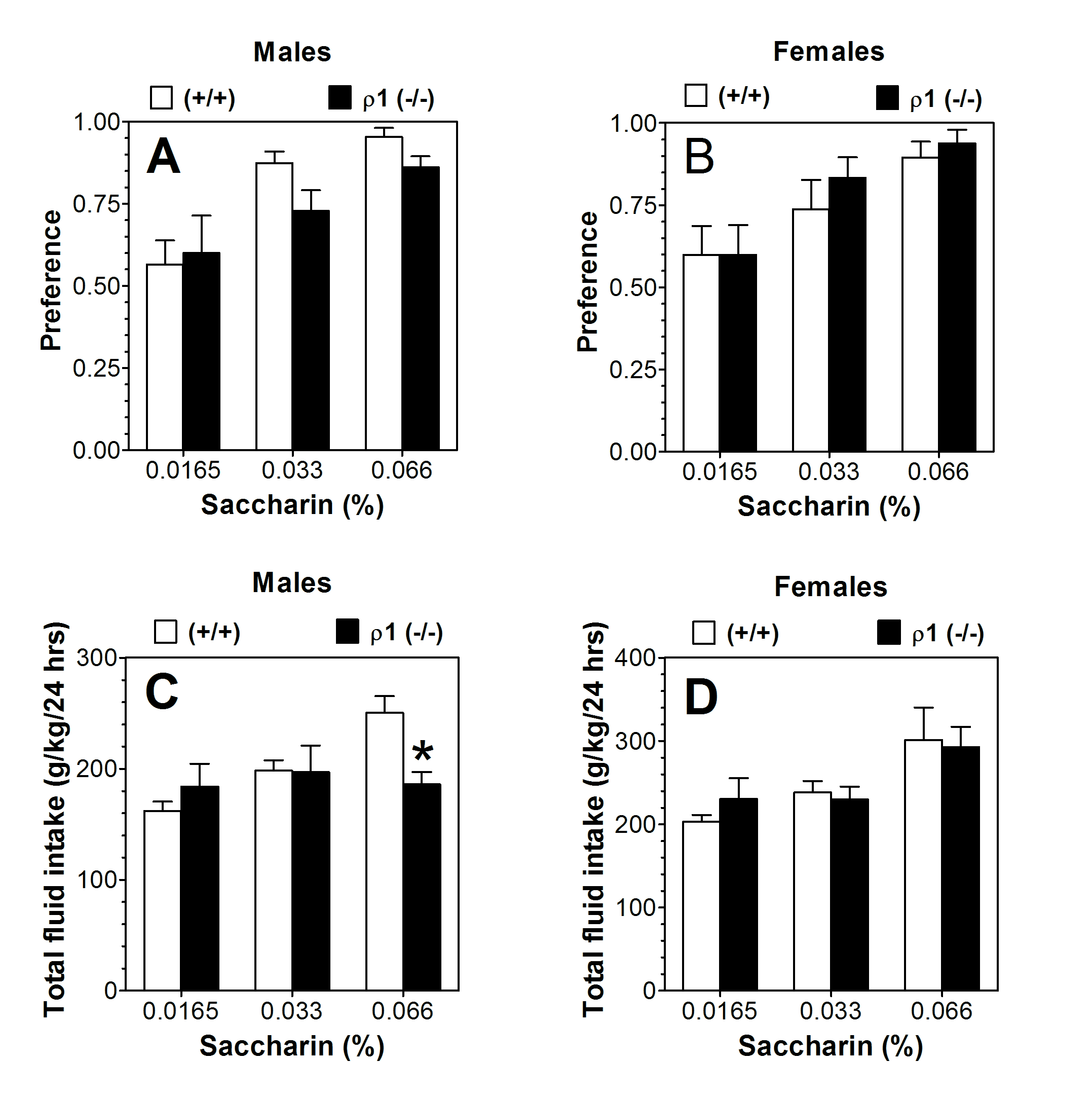

Supplement: Figure S1 — Voluntary saccharin consumption was not different between ρ1 (−/−) and wild type mice in two-bottle choice paradigm. A. Preference for saccharin in males. (F2,36 = 22, p<0.001, main effect of concentration; no main effect of genotype or genotype x concentration interaction). B. Preference for saccharin in females. (F2,32 = 34.1, p<0.001, main effect of concentration; no main effect of genotype or genotype x concentration interaction). C. Total fluid intake in males. (F2,36 = 7.8, p<0.01, main effect of genotype; F2,36 = 7.8, p<0.01, genotype x concentration interaction; no main effect of genotype). *p<0.05 vs. corresponding wild type mice for the same concentration of saccharin. D. Total fluid intake in females. (F2,32 = 8.6, p<0.01, main effect of concentration; no main effect of genotype or genotype x concentration interaction). Values represent mean ± S.E.M. Data were analyzed by two-way ANOVA with repeated measures with Bonferroni post hoc test (n = 8–10 per genotype for both sexes). ρ1 (−/−) = ρ1 null mice; (+/+) = wild type mice. (TIFF) [file pone.0085525.s001.tiff]

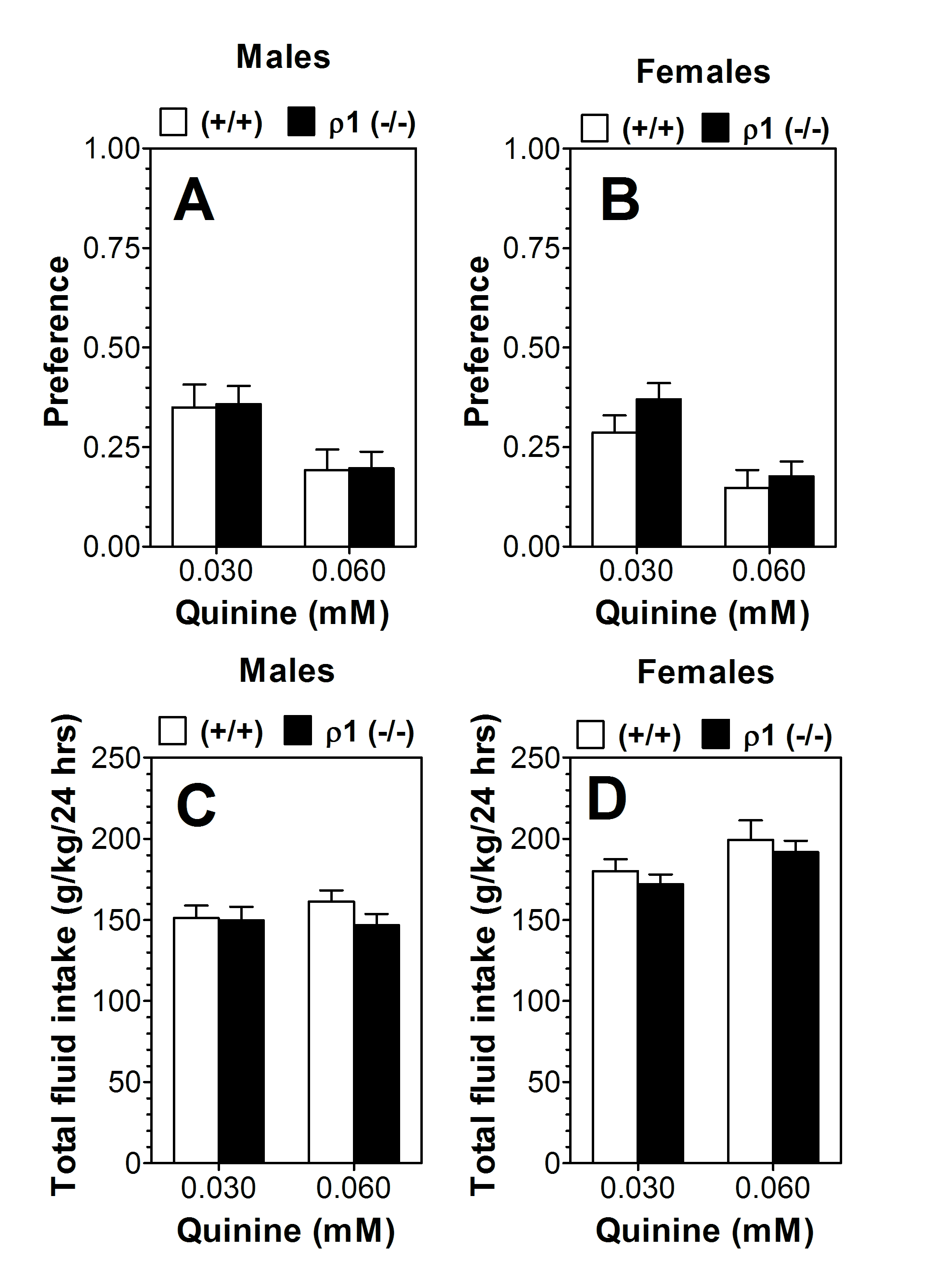

Supplement: Figure S2 — Voluntary quinine consumption was not different for ρ1 (−/−) and wild type mice in two-bottle choice paradigm. A. Preference for quinine in males. (F1,18 = 47.4, p<0.001, main effect of concentration; no main effect of genotype or genotype x concentration interaction). B. Preference for quinine in females. (F1,17 = 70.7, p<0.001, main effect of concentration; no main effect of genotype or genotype x concentration interaction). C. Total fluid intake in males. No main effect of genotype, concentration or genotype x concentration interaction. D. Total fluid intake in females. (F1,17 = 16, p<0.001, main effect of concentration; no main effect of genotype or genotype x concentration interaction). Values represent mean ± S.E.M. Data were analyzed by two-way ANOVA with repeated measures with Bonferroni post hoc test (n = 8–10 per genotype for both sexes). ρ1 (−/−) = ρ1 null mice; (+/+) = wild type mice. (TIFF) [file pone.0085525.s002.tiff]

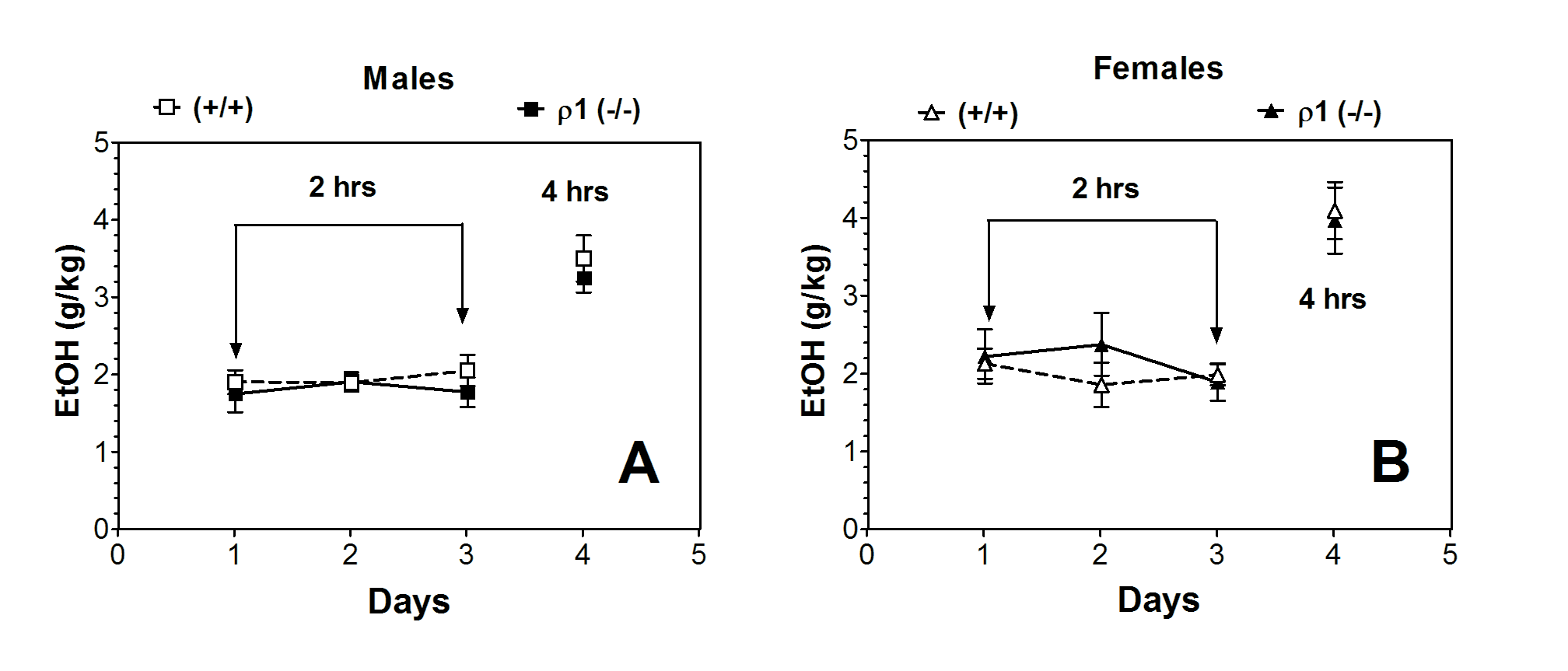

Supplement: Figure S3 — Ethanol intake in a limited access (one bottle DID) model was not different between ρ1 (−/−) and wild type mice. The amount of ethanol consumed (g/kg) with either 2- or 4-hour access periods is shown. A. Male mice (n = 8–11 per genotype). B. Female mice (n = 7–9 per genotype). No main effect of genotype, concentration or genotype x concentration interaction for the 2-hour access period; no difference in ethanol intake between the two genotypes for the 4-hour access period for either male or female mice (Student's t-test). Values represent mean ± S.E.M. ρ1 (−/−) = ρ1 null mice; (+/+) = wild type mice; EtOH = ethanol. (TIFF) [file pone.0085525.s003.tiff]

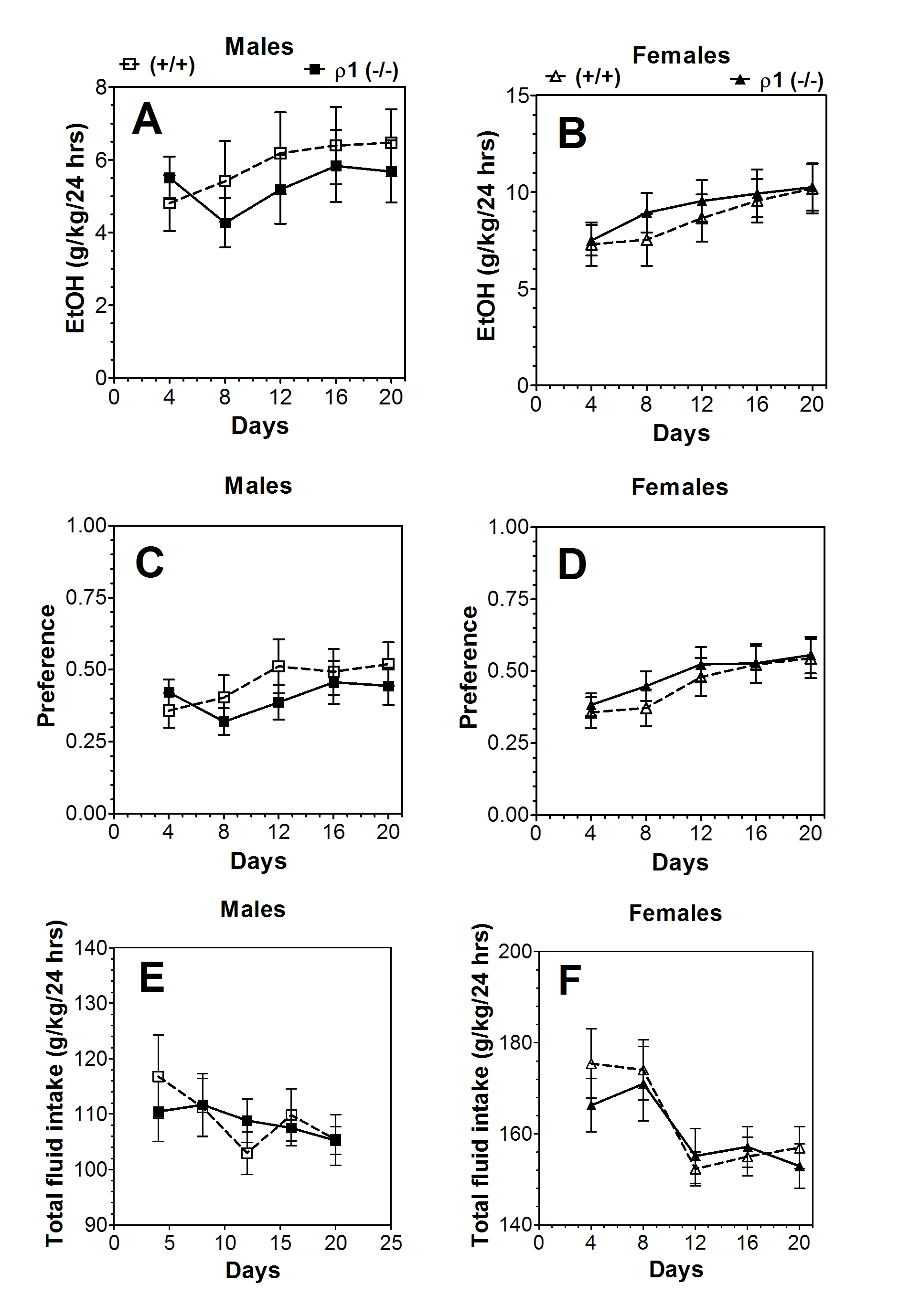

Supplement: Figure S4 — Ethanol intake in a two-bottle choice test with intermittent access to ethanol (every other day drinking) was not different between ρ1 (−/−) and wild type mice. A. Ethanol consumed (g/kg/24 hr) in males. (F4,80 = 3.2, p<0.05, main effect of time). B. Ethanol consumed (g/kg/24 hr) in females. (F4,132 = 8.5, p<0.001 main effect of concentration). C. Preference for ethanol in males. (F4,80 = 4.1, p<0.01, main effect of concentration). D. Preference for ethanol in females. (F4,132 = 14.6, p<0.001, main effect of concentration). E. Total fluid intake (g/kg/24 hr) in males. (F4,80 = 2.8, p<0.05, main effect of concentration). F. Total fluid intake (g/kg/24 hr) in females. (F4,132 = 13.4, p<0.001, main effect of concentration). No main effect of concentration or genotype x concentration interaction was found for any of the groups. Values represent mean ± S.E.M. Data were analyzed by two-way ANOVA with repeated measures with Bonferroni post hoc test (n = 9–10 per genotype for both sexes). ρ1 (−/−) = ρ1 null mice; (+/+) = wild type mice; EtOH = ethanol. (TIFF) [file pone.0085525.s004.tiff]

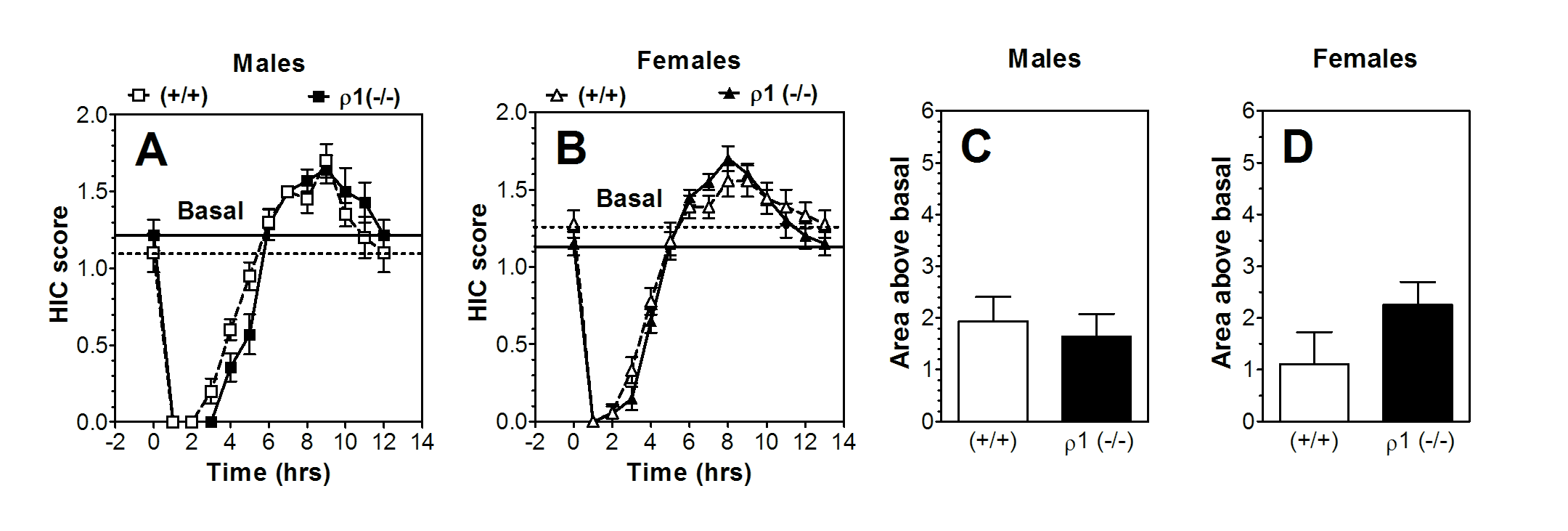

Supplement: Figure S5 — Severity of acute ethanol-induced withdrawal was not different between ρ1 (−/−) and wild type mice. A. Males, HIC score. B. Females, HIC score. C. Males, Area under the HIC score and above the basal level. D. Females, Area under the HIC score and above the basal level. No differences between the two genotypes were found for either male or female mice (Student's t-test). Values represent mean ± S.E.M. (n = 7–10 for male and n = 9–10 for female mice of both genotypes). ρ1 (−/−) = ρ1 null mice; (+/+) = wild type mice; HIC = handling induced convulsions. (TIFF) [file pone.0085525.s005.tiff]

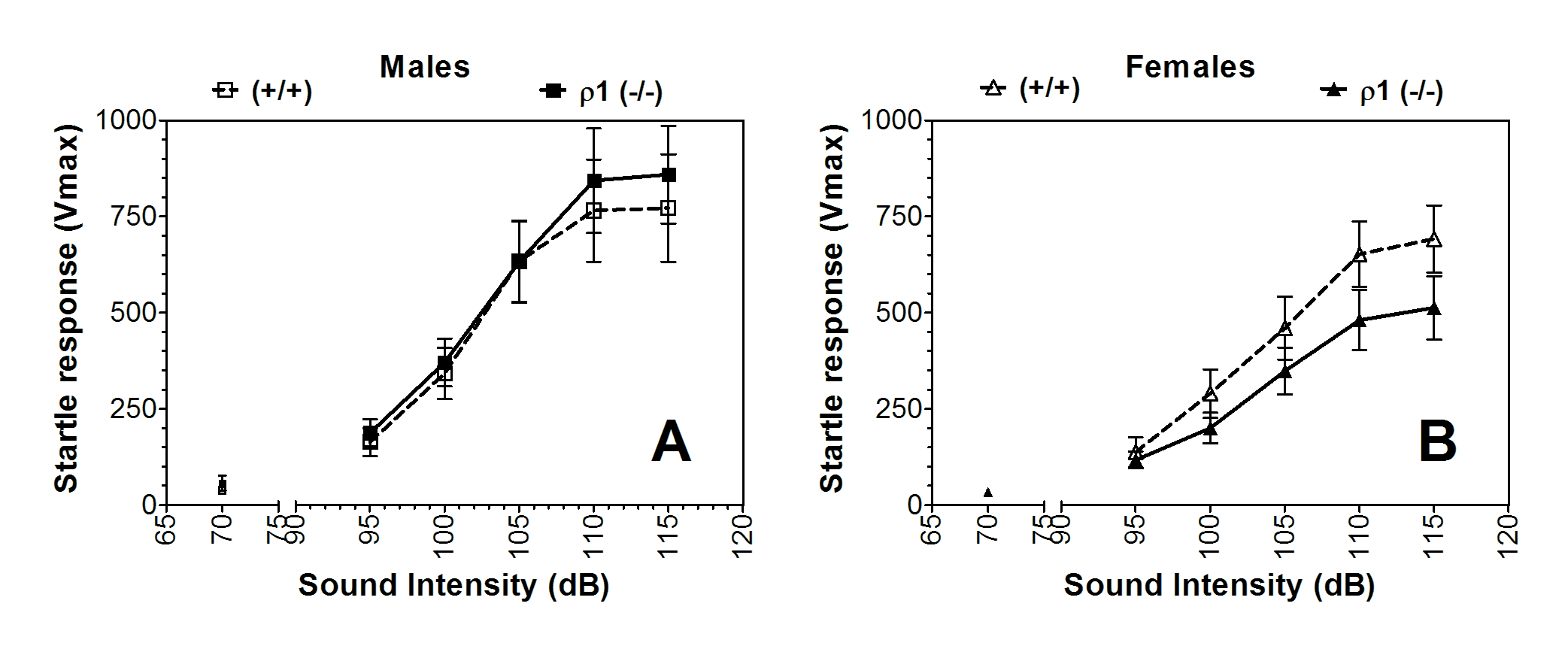

Supplement: Figure S6 — Acoustic startle response is not changed in ρ1 (−/−) mice of either sex. Data represent the maximum startle amplitude (Vmax) as a function of sound intensity (decibels). A. Males (n = 9–10 per genotype; F4,68 = 50; p<0.001, main effect of sound intensity). B. Females (n = 12–19 per genotype; F4,116 = 79.6; p<0.001, main effect of sound intensity). No main effect of genotype or genotype x sound intensity interaction was found for either male or female mice. Values represent mean ± S.E.M. Data were analyzed by two-way ANOVA with repeated measures with Bonferroni post hoc test. ρ1 (−/−) = ρ1 null mice; (+/+) = wild type mice. (TIFF) [file pone.0085525.s006.tiff]

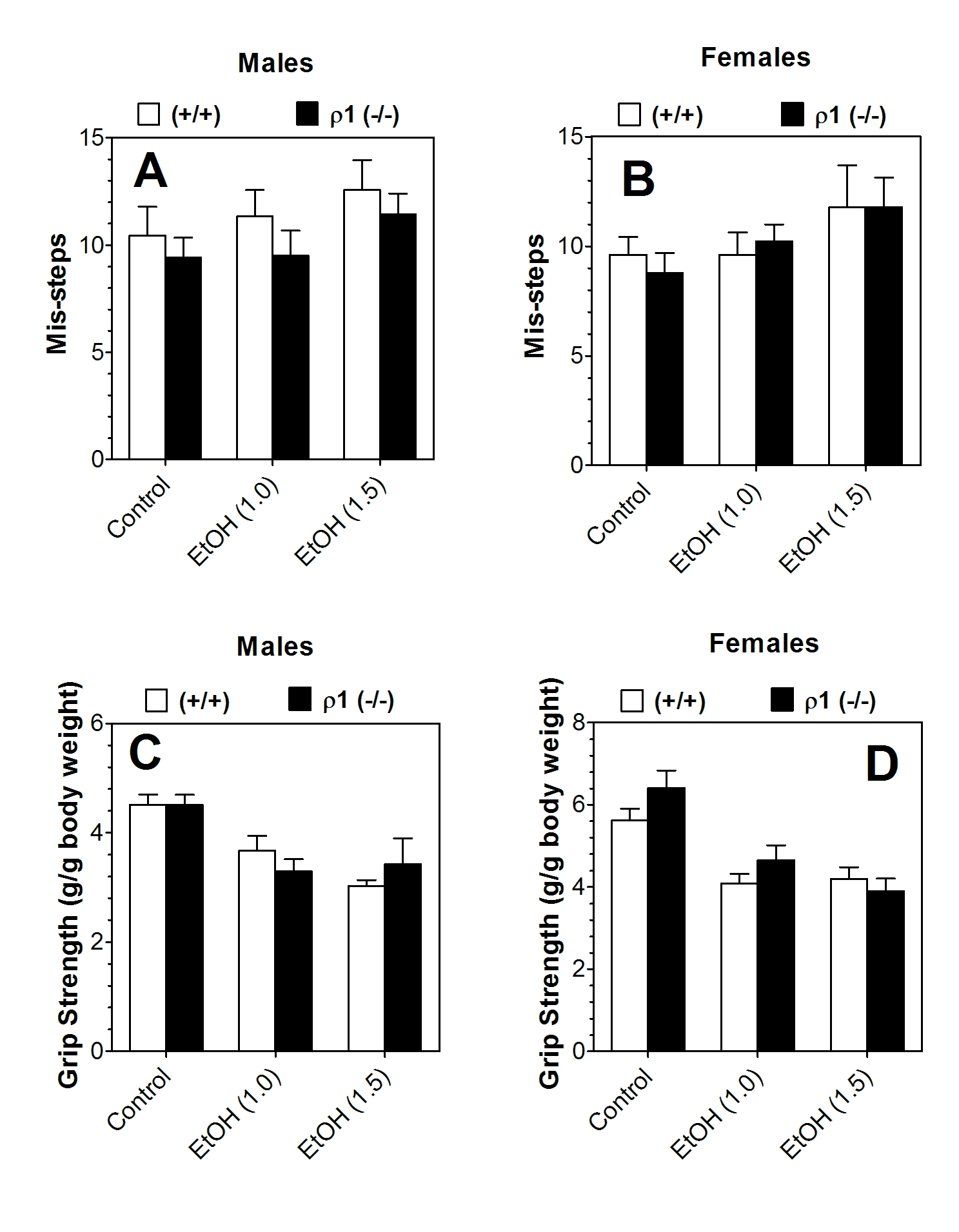

Supplement: Figure S7 — Ethanol produced similar alteration in grip strength and number of missteps in wild type and ρ1 (−/−) mice. A. Number of missteps in males (n = 7–9 per genotype). B. Number of missteps in females (n = 7–9 per genotype). No dependence on genotype, dose or genotype x dose interaction was found for either male or female mice. C. Grip strength in males (n = 7–9 per genotype; F2,28 = 18.1; p<0.001, dependence on dose). D. Grip strength in females (n = 7–9 per genotype; F2,28 = 42; p<0.001, dependence on dose). No dependence on genotype or genotype x dose interaction was found for either male or female mice. Values represent mean ± S.E.M. Data were analyzed by two-way ANOVA with repeated measures with Bonferroni post hoc test. ρ1 (−/−) = ρ1 null mice; (+/+) = wild type mice; EtOH = ethanol. (TIFF) [file pone.0085525.s007.tiff]
